# Interlaboratory validation of an optimized protocol for measuring α-amylase activity by the INFOGEST international research network

**DOI:** 10.1038/s41598-025-12561-y

**Published:** 2025-08-23

**Authors:** Daniela Freitas, Shannon Gwala, Gwénaële Henry, Athina Lazaridou, Christine Boesch, Dorine Duijsens, Faye Wheller, Ivan M. Lopez-Rodulfo, Kali Kotsiou, Kendall R. Corbin, Marilisa Alongi, Mario M. Martinez, Maryam S. Hafiz, Monic M. M. Tomassen, Natalia Perez-Moral, Natalia P. Vidal, Renata M. C. Ariëns, Sebnem Simsek, Sedef Nehir El, Sibel Karakaya, Steven Le Feunteun, Shanna Bastiaan-Net, Svenja Krause, Bin Zhang, Caroline Orfila, Simon Ballance, Terri Grassby

**Affiliations:** 1 Teagasc Food Research Centre, Moorepark, Fermoy, P61 C996 County Cork Ireland; 2 INRAE, Institut Agro, STLO, 35042 Rennes, France; 3 Laboratory of Food Chemistry and Biochemistry, Department of Food Science and Technology, School of Agriculture, Aristotle University of Thessaloniki, P.O. Box 235, 54124 Thessaloniki, Greece; 4 School of Food Science and Nutrition, University of Leeds, Leeds, United Kingdom; 5 Laboratory of Food Technology, Department of Microbial and Molecular Systems (M2S), KU Leuven, Kasteelpark Arenberg 23, PB 2457, 3001 Leuven, Belgium; 6 School of Biosciences, Faculty of Health and Medical Sciences, University of Surrey, Guildford, GU2 7XH United Kingdom; 7 Center for Innovative Food (CiFOOD), Department of Food Science, Aarhus University, Agro Food Park 48, N 8200 Aarhus, Denmark; 8 Department of Horticulture, Martin-Gatton College of Agriculture, Food and Environment, University of Kentucky, Lexington, KY United States of America; 9 Department of Agricultural, Food, Environmental and Animal Sciences, University of Udine, Udine, Italy; 10 Food Technology Area, Department of Agricultural Engineering, University of Valladolid, Valladolid, Spain; 11 Department of Clinical Nutrition, Faculty of Applied Medical Sciences, King Abdulaziz University, Jeddah, Saudi Arabia; 12 Wageningen Food & Biobased Research, Wageningen University & Research, 6708 WG Wageningen, The Netherlands; 13 Quadram Institute Bioscience, Rosalind Franklin Road, Norwich Research Park, Norwich, NR4 7UQ United Kingdom; 14 Department of Food Engineering, Faculty of Engineering, Ege University, 35100 İzmir, Türkiye; 15 Global Oatly Science and Innovation Centre, Rydbergs Torg 11, Space Building, Science Village, 22 484 Lund, Sweden; 16 School of Food Science and Engineering, South China University of Technology, Guangzhou, 510640 China; 17 Fisheries and Aquaculture Research, Nofima AS, Norwegian Institute of Food, PB 210, N-1433 Ås, Norway

**Keywords:** Biochemistry, Biological techniques

## Abstract

The activity of α-amylases is frequently determined using a single-point assay at 20 °C. Previous work within INFOGEST “Working Group 5 - Starch digestion and amylases” identified significant interlaboratory variation with this protocol. The current study aimed to evaluate the repeatability (intralaboratory precision) and reproducibility (interlaboratory precision), measured as coefficients of variation (CVs), of a newly optimized protocol version based on four time-point measurements at 37 °C. Human saliva (a pool from ten healthy adults) and three porcine enzyme preparations (two pancreatic α-amylases and pancreatin) were tested in 13 laboratories across 12 countries and 3 continents. Assay repeatability for each lab remained below 20% for all test products and the overall repeatability was below 15%, ranging between 8 and 13% for all products. Reproducibility was greatly improved with interlaboratory CVs ranging from 16 to 21%, *i.e.* up to four times lower than with the original method. Five laboratories repeated the same assay at 20 °C, and the amylolytic activity of each product increased by 3.3-fold (± 0.3) from 20 to 37 °C. The newly optimized protocol is henceforth recommended to ensure precise determinations of α-amylase activity levels and to facilitate comparisons across different studies.

## Introduction

Food digestion is a central element of human health, one that is strongly dependent on, and influenced by, enzymatic action. Up to 50% of our energy intake can be derived from a single carbohydrate, starch^1^. α-Amylases (EC 3.2.1.1) of salivary and pancreatic origin play a key role in the digestive process of starch and related physiological implications^2^. Therefore, the accurate characterization of these digestive enzymes is a pre-requisite for investigating and understanding starch digestion in human, animal, and in vitro studies. In clinical laboratories, the measurement of α-amylase, and specifically pancreatic α-amylase, in human serum and urine is a well-established and routinely used marker of acute pancreatitis – the main clinical application of this enzyme since the first description of its diagnostic value in 1929^3^. In addition to this, α-amylase activity determinations can also be used as proxy measures of other outcomes of interest in both research and clinical settings. Salivary α-amylase, for example, has been studied as a non-invasive biomarker of stress^4^, as well as a potential discriminating marker^5^ and indicator of glycaemic control^6^ in type-II diabetes. Pancreatic α-amylase, on the other hand, has been studied as a potential indicator of anastomotic leakage, which is one of the most common complications following intestinal surgery^7,8^.

Benchtop assays for determining the activity of salivary or pancreatic α-amylase of human or porcine origin are advantageous due to the low cost, ease of set-up and relatively fast turnaround. However, a major drawback in this field of research is that there are numerous different assays currently in use, making it extremely difficult to confidently compare results across different studies.

Collaborative work within INFOGEST, an international research network of over 700 scientists from 200 institutes in 52 countries, has led to the development of harmonised protocols for the study of digestion^9–11^. The conditions in these protocols have been carefully defined to ensure their physiological relevance. They include recommendations on enzyme characterisation assays to be carried out in preparation for digestion studies as well as target levels of enzymatic activity during in vitro experiments. The recommended assay to measure α-amylase activity in fluids and enzyme preparations of human or animal origin while setting up INFOGEST in vitro digestion protocols was first described by Bernfeld in 1955^9,12^. It consists of a single-point measurement whereby the reducing sugars formed during the incubation (3 min at 20 °C) of a potato starch solution (substrate) with α-amylase are quantified as maltose equivalents. The resulting definition of α-amylase activity units is as follows: one unit liberates 1.0 mg of maltose from starch in 3 min at pH 6.9 at 20 °C. This assay has been extensively used over the past decades across a wide range of knowledge areas with publications in the fields of “Biochemistry, Genetics and Molecular Biology”, “Agricultural and Biological Sciences” and “Immunology and Microbiology” making up more than half of the citations^13^. However, no interlaboratory comparison studies of its performance have yet been carried out. Preliminary tests undertaken by the INFOGEST “Working Group 5 - Starch digestion and amylases” revealed that there can be large variations in the results obtained by different labs, with reproducibility coefficients of variation (CV_R_) up to 87%^14^. Continued efforts within the working group to optimize this protocol have culminated in the development of a new version of the assay with the main changes pertaining to the incubation conditions (temperature and duration) and number of sampling points, as well as recommendations for the preparation of the assay solutions. In this context, the goal of this interlaboratory study was to test this new protocol and to evaluate its repeatability and reproducibility, measured as intralaboratory and interlaboratory coefficients of variation (CV_r_ and CV_R_, respectively). Each participating laboratory tested the same set of chemicals using a variety of laboratory instruments requiring specific experimental set-up variations as described in the next section. The setup of this ring trial is presented and the results, and performance of the newly optimized protocol are discussed.

## Results

### Protocol implementation at each laboratory

The newly optimized protocol was successfully implemented by all the laboratories participating in the ring trial (n = 13) using their own equipment. Four different commercial enzyme preparations were tested by each lab: two porcine pancreatic α-amylase samples (each from a different supplier, referred to as α-amylase M and α-amylase S), porcine pancreatin and human saliva. All labs analysed the test products at the recommended concentrations (Table S7 – Supplementary material). The main differences in terms of protocol implementation were related to the instruments used for the incubations (water bath with or without shaking *vs.* thermal shaker), and spectrophotometry (spectrophotometer *vs.* microplate reader). A detailed list of the equipment used for these two protocol steps is presented in Table S6 (Supplementary material).

### Calibrators and calibration curves

Each lab received a stock maltose solution (2%, w/v) for the preparation of ten calibrator solutions (concentration range 0–3 mg/mL, Table S2 in the Supplementary material). The equations of the calibration curves were established through linear regression. The calibrations were performed one to four times, depending on the laboratory. A total of 32 calibration curves have been reported (Table S8, Supplementary material), 22 of them were established using a microplate reader and the remaining were obtained using a cuvette format spectrophotometer. Reproducibility (CV_R_) was similar irrespective of the format used (23% and 27% for microplate and cuvettes, respectively). All calibration curves showed a high linearity (r^2^ between 0.98 and 1.00) with a global r^2^ of 1.00.

### Assay performance

The definition of α-amylase activity resulting from the application of the newly optimized protocol to measure α-amylase activity is the following:

- Based on the definition originally proposed by Bernfeld: one unit liberates 1.0 mg of maltose equivalents from potato starch in 3 minutes at pH 6.9 at 37°C.
- Based on the international enzyme unit (IU) definition standards: one unit liberates 1.0 μmol of maltose equivalents from potato starch in 1 minute at pH 6.9 at 37°C.

Conversions between these two unit definitions can be made as follows: 1 Bernfeld unit = 0.97 IU.

**Fig. 1:**
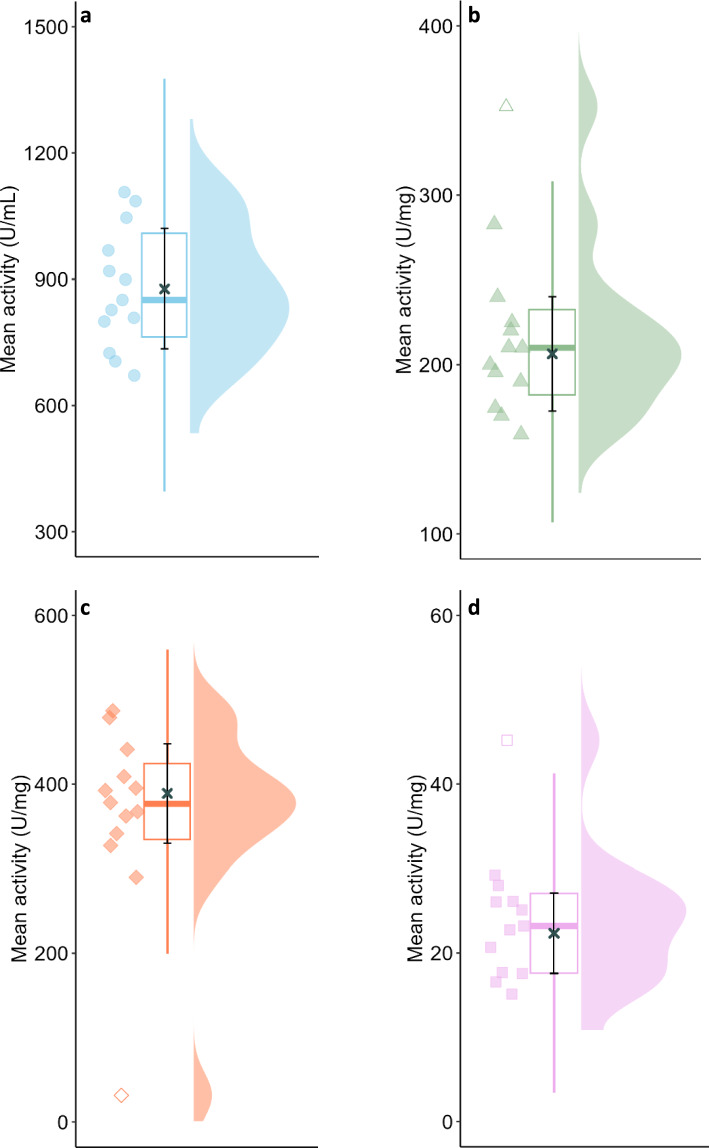
Variations in mean enzymatic activities across 13 laboratories using the newly optimized protocol at 37 °C. Samples: (**a**) human saliva, (**b**) porcine pancreatin, (**c**) porcine α-amylase M, and (**d**) porcine α-amylase S. Scatter plots (markers next to the y-axes) show individual lab means. On the opposite end of the graphs, the half-violin plots (filled curves) represent the data distribution. Box plots: median (thick lines), interquartile range (box), 1.5 × interquartile range (whiskers). Outliers are indicated with open markers on the scatter plots. Black “x” symbols and lines over the boxplots: mean results of all labs and corresponding standard deviations (excluding outliers).

The results reported by all the laboratories participating in the ring trial are presented in (Table 1 (Panel A)). The variation in the results obtained at 37 °C is depicted in (Fig. 1). All thirteen laboratories measured α-amylase activity in the four samples supplied, at three different concentrations. The results obtained for the analysis of human saliva, porcine pancreatin, α-amylase M, and α-amylase S are presented in panels (a), (b), (c) and (d) of Fig. 1, respectively. The scatter plots (markers next to the y-axes) show the mean activities reported by each lab. On the opposite end of the graphs, the half-violin plots (filled curves) represent the data distribution. In the box plots, the boxes hold 50% of the data, with an equal number of data points above and below the median (thick coloured lines) and the whiskers (coloured vertical lines) indicate the range of data falling within 1.5 × box length beyond the upper and lower limits of the box. Outliers beyond this range are indicated with open markers on the scatter plots. The × symbol on each graph marks the mean results of all labs and the corresponding standard deviations are represented by the capped black lines (these calculations excluded outlier data points).

**Table 1:** Overview of the results.

	α-amylase M								α-amylase S								Pancreatin								Human saliva							
	20 °C				37 °C				20 °C				37 °C				20 °C				37 °C				20 °C				37 °C			
	Activity (Mean ± SD)			CV (%)	Activity (Mean ± SD)			CV (%)	Activity (Mean ± SD)			CV (%)	Activity (Mean ± SD)			CV (%)	Activity (Mean ± SD)			CV (%)	Activity (Mean ± SD)			CV (%)	Activity (Mean ± SD)			CV (%)	Activity (Mean ± SD)			CV (%)
Panel A – Amylase activities^1^ as reported by each participating lab																																
Lab A	116.5	±	8.5	7.3	487.3	±	48.4	9.9	7.9	±	0.2	2.2	28.0	±	4.3	15.4	68.1	±	7.6	11.1	240.1	±	23.8	9.9	184.9	±	15.4	8.3	828.4	±	97.5	11.8
Lab B	107.3	±	12.7	11.8	340.6	±	11.5	3.4	5.5	±	0.3	5.3	17.7	±	0.5	3.0	57.3	±	1.5	2.6	169.8	±	7.6	4.5	271.8	±	12.7	4.7	702.9	±	72.8	10.4
Lab C	122.7	±	6.7	5.5	409.0	±	51.7	12.6	7.9	±	0.9	10.8	22.7	±	1.9	8.3	58.8	±	6.4	10.8	210.3	±	23.4	11.1	299.5	±	29.4	9.8	851.1	±	141.1	16.6
Lab D	67.3	±	15.5	22.9	289.4	±	10.6	3.7	5.4	±	0.5	8.8	17.6	±	0.9	5.2	56.5	±	8.6	15.2	158.8	±	12.5	7.8	212.0	±	26.7	12.6	669.7	±	89.0	13.3
Lab E	105.8	±	15.9	15.0	376.7	±	57.3	15.2	6.3	±	1.0	15.3	16.6	±	2.9	17.4	53.7	±	6.2	11.5	174.5	±	23.4	13.4	298.2	±	33.6	11.3	914.4	±	133.1	14.6
Lab F	–				395.6	±	44.7	11.3	–				25.1	±	2.4	9.5	–				190.1	±	2.9	1.5	–				809.8	±	59.5	7.3
Lab G	–				366.6	±	14.7	4.0	–				23.2	±	1.2	5.3	–				210.1	±	4.7	2.25	–				1082.6	±	109.9	10.2
Lab H	–				439.9	±	41.0	9.3	–				26.1	±	1.5	5.8	–				225.0	±	9.0	3.99	–				895.4	±	95.2	10.6
Lab I	–				363.0	±	25.2	7.0	–				15.1	±	0.2	1.0	–				220.3	±	27.0	12.3	–				1109.0	±	201.6	18.2
Lab J	–				392.0	±	39.1	10.0	–				20.7	±	1.5	7.4	–				200.0	±	4.0	2	–				798.7	±	54.6	6.8
Lab K	–				328.3	±	39.5	12.0	–				26.1	±	1.3	5.0	-				195.7	±	10.7	5.5	–				728.1	±	18.8	2.6
Lab L	–				479.5	±	61.4	12.8	–				29.2	±	2.6	8.9	–				282.8	±	12.4	4.4	–				970.1	±	170.1	17.5
Lab M	–				31.5	±	0.5^2^	1.7^2^	–				45.2	±	4.4^2^	9.7^2^	–				352.3	±	45.3^2^	12.9^2^	–				1046.7	±	61.8	5.9
Activity (Mean ± SD)	**103.7**^**X**^** ± 21.4**				**389.0**^**B**^** ± 58.9**				**6.6**^**Z**^** ± 1.2**				**22.3**^**D**^** ± 4.8**				**58.9**^**Y**^** ± 5.5**				**206.5**^**C**^** ± 33.8**				**253.3**^**W**^** ± 52.1**				**877.4**^**A**^** ± 142.7**			
*Activity in IU*^*3*^* (Mean* ± *SD)*					*377.3* ± *57.1*								*21.6* ± *4.7*								*200.3* ± *32.8*								*851.1* ± *138.4*			
Panel B – Assay repeatability and reproducibility																																
Repeatability (CV_*r*_, *%*)	14.0				10.0				9.6				8.9				11.0				7.7				9.7				12.5			
Reproducibility (CV_*R*_,* %*)	20.6				15.1				18.8				21.3				9.3				16.4				20.6				16.3			
Panel C – Relationship between activities measured at 37 °C vs. 20 °C																																
Ratio of activity at 37 °C *vs.* 20 °C (Mean ± SD)^4^	3.7 ± 0.5								3.1 ± 0.4								3.2 ± 0.3								3.2 ± 0.7							
Global average^5^ of the activity ratios between 37 °C and 20 °C = 3.3 ± 0.3 (95% CI: 2.9 to 3.7)																																

^1^Amylase activity unit definitions: assay at 20 °C—one unit liberates 1.0 mg of maltose from starch in 3 min at pH 6.9 and 20 °C; assay at 37 °C—one unit liberates 1.0 mg of maltose from starch in 3 min at pH 6.9 and 37 °C.^2^Excluded from calculation of activity mean and SD, CV_*r*_, CV_*R*_ and from the statistical analysis. ^3^Conversion of mean α-amylase activities obtained using the newly optimized protocol (at 37 °C) into International Units (IU) by multiplying activity values based on Bernfeld’s original definition by the conversion factor 0.97. ^4^Average of the ratios of activity at 37 °C vs. 20 °C reported by laboratories A to E for each test product. ^5^Average of the ratios of activity at 37 °C vs. 20 °C of all test products as reported by laboratories A to E.

^A–D^Within a row, denote statistically significant differences between the products tested at 37 °C (p < 0.0001). ^W-Z^ Within a row, denote statistically significant differences between the products tested at 20 °C (p < 0.0001).

Overall, 5.8% of data was identified as an outlier (three out of 52 data points). By analysing Fig. 1 without considering the outlier data points, it is possible to observe that, overall, the data was normally distributed. This was reflected in the good agreement between the median and mean activities of each test product. The mean activities of saliva, pancreatin, α-amylase M and α-amylase S were 877.4 ± 142.7 U/mL, 206.5 ± 33.8 U/mg, 389 ± 58.9 U/mg and 22.3 ± 4.8 U/mg (mean ± SD), respectively. The difference between the α-amylase activities in all products tested was statistically significant (p < 0.0001).

The incubation of the enzyme solutions with the substrate was a critical step of the protocol for which there were variations in the experimental set ups used (Table S6, Supplementary material). To investigate whether this impacted the results, the type of incubations performed, *i.e.* water bath with or without shaking *vs.* thermal shaker were also considered in the statistical analyses of the results. No significant effect of incubation conditions nor of their interaction with the type of product or concentrations tested has been identified (p > 0.05).

**Fig. 2:**
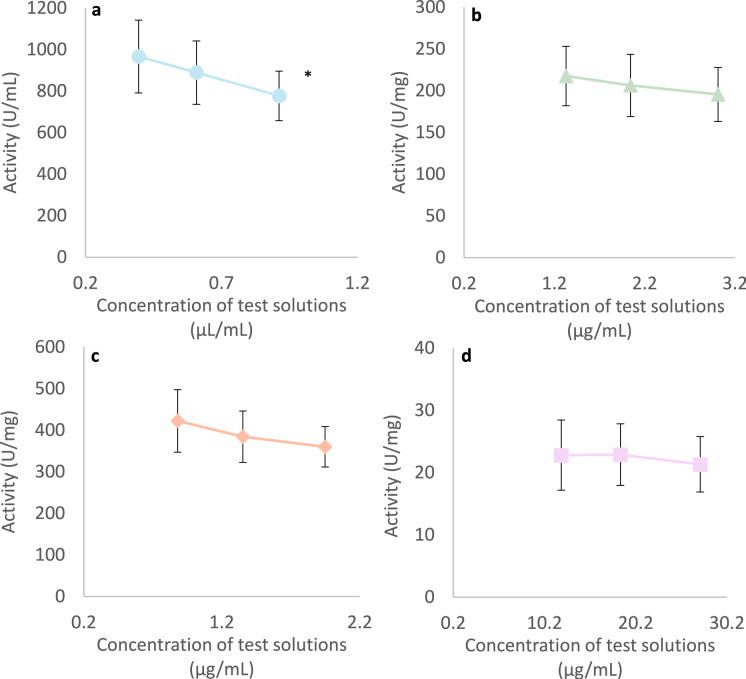
Amylolytic activity as a function of enzyme concentration (C1, C2 and C3). Each data point represents the mean ± SD of 13 laboratories for (**a**) saliva, and of 12 laboratories for (**b**) pancreatin, (**c**) α-amylase M and (**d**) α-amylase S.

Each product has been tested at three different concentrations (referred to as solutions C1, C2 and C3 in the Methods’ section “α-amylase solutions”). Mean α-amylase activities, calculated as a function of the concentrations of the test products in the solutions used during the incubations are represented in (Fig. 2). There were no statistically significant differences (p > 0.05) between the results obtained at the different concentrations of pancreatin, α-amylase M, and α-amylase S. For saliva, there was a statistically significant difference between the results obtained with the lowest and highest concentrations (p = 0.01).

**Fig. 3:**
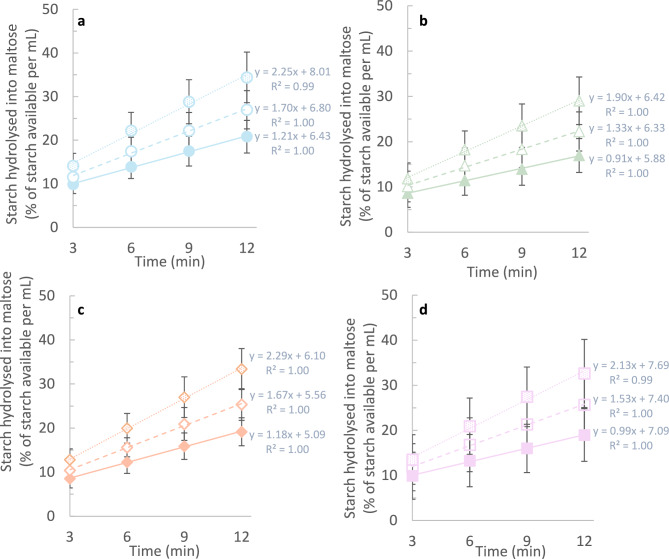
Starch hydrolysis kinetics. Reducing sugars measured as maltose equivalents following incubations with three different concentrations of each enzyme (C1, C2 and C3) were converted into starch (conversion factor of 0.95). The result is expressed as the proportion of total starch (available at t_0_) hydrolysed over time. Each data point represents the mean ± SD of 13 laboratories for (**a**) saliva, and of 12 laboratories for (**b**) pancreatin, (**c**) α-amylase M and (**d**) α-amylase S.

To estimate substrate availability during incubations for each concentration tested, the release of reducing sugars (in maltose equivalents) has been converted into starch (multiplication by the theoretical maltose to starch conversion factor 0.95) and expressed as percentage of total starch (*i.e.* starch available at t = 0 min) and plotted against time (min) (Fig. 3). After 12 min of incubation, the proportion of total maltose released, remained equivalent to < 35% of the total starch available for all products at all concentrations tested. To further examine the linearity of the enzyme reactions, the *r*^*2*^ of the linear regression curves fitted to these data sets have been computed. All curves obtained by each lab with each test product were linear. The global *r*^*2*^ (average of all labs) varied between 0.99 and 1.00 for all products depending on the concentration tested.

**Fig. 4:**
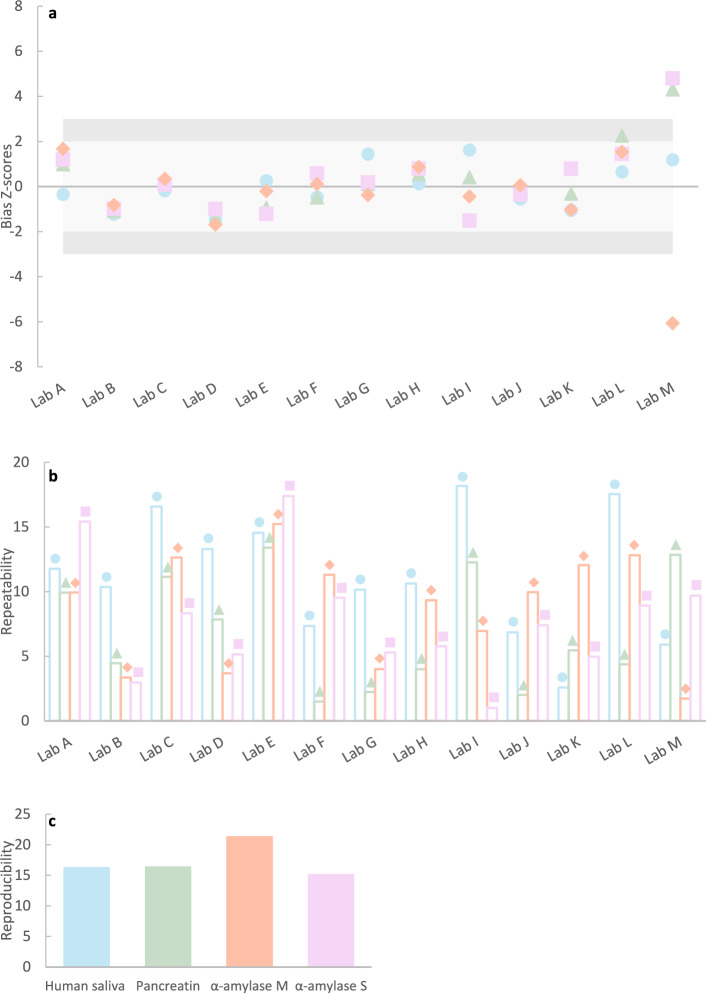
Method performance across laboratories and products. (**a**) Z-score distribution of each laboratory’s bias relative to the mean of all laboratories (excluding outlier values) for each product assayed at 37 °C. Each point represents one product by an individual laboratory. Horizontal line indicates reference (z = 0), the highlighted area where |z|≤ 2 indicates satisfactory performance, areas where |z|≥ 2 and |z|≤ 3 indicate unsatisfactory performance. (**b**) Repeatability (CV_r_, %) for each laboratory and product. (**c**) Reproducibility (CV_R_, %) among all laboratories, excluding outliers, for each product. Colour/Marker shape coding: blue/circle – saliva; green/triangle – Porcine pancreatin; orange/diamond—α-amylase M; pink – α-amylase S.

Interlaboratory bias scores were calculated for each product across 13 participating laboratories and converted into z-scores to enable a standardized assessment of systematic errors. Z-score analysis (Fig. 4a) revealed that over 90% of the results (48 out of 52) were within the satisfactory range, with a Z-score between − 2 and 2. Three results were considered unsatisfactory as they exceeded the critical value of |3|, and they coincided with the outlier data points identified earlier. One additional result had a z-score of 2.3. Original bias scores are provided in the supplementary material (Fig. S3).

Assay repeatability (CV) for each lab remained below 20% for all test products (Table 1-Panel A, illustrated in Fig. 4b). The overall repeatability of the method (CV_r_) for each product tested was below 15% (Table 1—Panel B). Overall repeatability and intralaboratory CV ranges (CV_r_ [min CV—max CV]) for saliva, pancreatin, α-amylase M, and α-amylase S were 12.5% [2.6–18.2%], 7.7% [1.5–13.4%], 10% [3.4–15.2%] and 8.9% [1.0–17.4%], respectively.

Reproducibility (CV_R_) remained below 20% for three out of the four products tested: saliva (16.3%), pancreatin (16.4%) and α-amylase M (15.1%) (Table 1 – Panel C, Fig. 4c). The highest CV_R_ was observed with α-amylase S (21.3%).

### Comparisons between the incubations at 20 and 37 °C

**Fig. 5:**
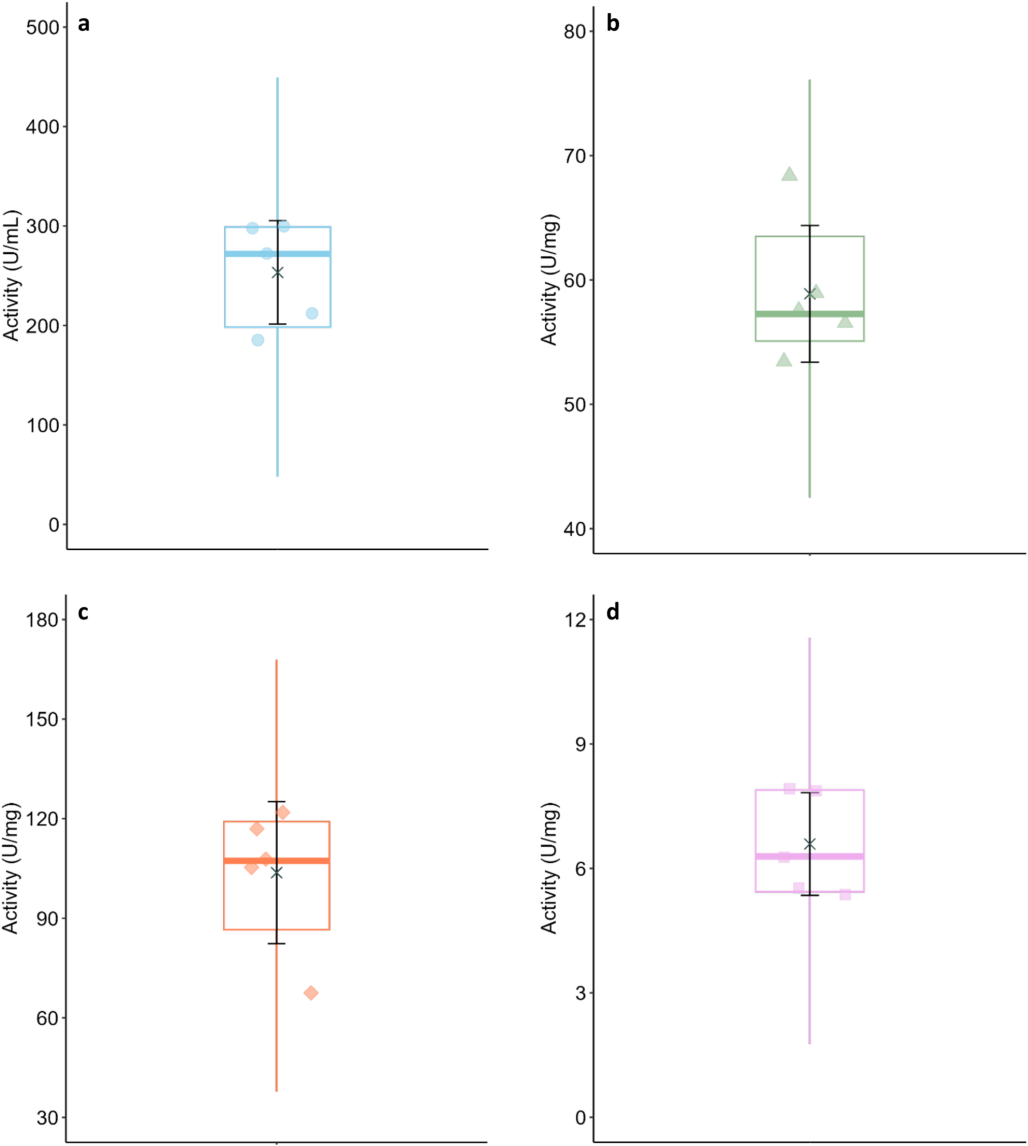
Variations in mean enzymatic activities at 20 °C across a subgroup of 5 laboratories. Samples: (**a**) saliva, (**b**) pancreatin, (**c**) α-amylase M and (**d**) α-amylase S. Scatter plots show individual lab means. Box plots: median (thick lines), interquartile range (box), 1.5 × interquartile range (whiskers). Black “x” symbols and lines over the boxplots: mean results of all labs and corresponding standard deviations (excluding outliers).

Guidelines on target α-amylase activity levels for in vitro digestion experiments are currently based on determinations carried out at 20°C^9^. To enable correlations with future α-amylase activity determinations using this optimized protocol, the possibility of deriving a mathematical relationship between α-amylase activities measured at 20 °C and 37 °C has also been investigated. For this purpose, the analysis of all samples has been repeated at 20 °C by a subgroup of five laboratories. The results are presented in (Fig. 5 and Table 1). The average α-amylase activities in saliva, pancreatin, α-amylase M and α-amylase S incubated at 20 °C were 253.3 ± 52.1 U/mL, 58.9 ± 5.5 U/mg, 104 ± 21.4 U/mL and 6.6 ± 1.2 U/mL, respectively. The differences between these results were statistically significant (p < 0.0001).

The ratios between the average α-amylase activities of each product at 37 °C *vs.* 20 °C are presented in Table 1 (Panel C). The average ratios for each product ranged between 3.1 ± 0.4 (α-amylase S) and 3.7 ± 0.5 (α-amylase M). One way ANOVA has been performed on this data, confirming that there were no statistically significant differences between the ratios obtained for each product (p > 0.05). Therefore, a global ratio of 3.3 ± 0.3 (95% CI: 2.9 to 3.7) has been determined by averaging the ratios from all four products.

## Discussion

The α-amylase activity assay described by Peter Bernfeld in 1955^12^ is widely used. Among its advantages, are the low cost and the fact that it is easily implementable in most laboratorial set ups. However, preliminary work with α-amylases M and S between eight laboratories of INFOGEST “Working Group 5—Starch digestion and amylases” revealed that, despite acceptable repeatability (CV_r_ between 13.0% and 18.2%), the previous version of the protocol had inadequate reproducibility (CV_R_ from 66.0% up to 87.3%)^14^. Continued efforts within this working group led to the newly optimized protocol presented here. This protocol has been tested by 13 laboratories (of which 7 had also participated in the preliminary studies mentioned above and previously described^14^).

In the current study, a low proportion of results (5.8%) have been identified as outliers, corresponding to one lab’s results for 3 out of the 4 tested products. These results were likely due to problems affecting analytical performance within the laboratory and were not attributable to the method itself. The outcome of the Z-score analysis, with over 90% of the results falling within the satisfactory range, demonstrated high consistency across laboratories and products. The reproducibility (CV_R_) of the slopes of the calibration curves ranged from 23 to 27%, depending on how the absorbance was measured. This variability encompasses variations in instrument responses to calibrators as well as differences in the preparation of solutions and general laboratory manipulation. As intended, the calibration curve captured an important part of instrument-related variability, resulting in lower variability for the enzymatic assays. The reproducibility coefficients of variation of the enzymatic assays of saliva, pancreatin and α-amylase M was between 15.1% and 16.4%. Only one product, α-amylase S, deviated from this range (CV_R_ = 21.3%). These results reflect a major improvement in reproducibility, placing the assay within the critical threshold of 30%, frequently considered an indicator of acceptable interlaboratory variability^15,16^. Additionally, the assay repeatability (at 37 °C) has also improved, as reflected by CV_r_ values which, on average, varied between 7.7% (pancreatin) and 12.5% (saliva).

Because there are several differences between the original assay and the current version of the protocol, it is not possible to point out a single factor responsible for this improvement. However, four modifications likely contributed significantly. Variability in the **preparation of enzyme solutions** was a key issue identified during protocol development, particularly for particulate samples like pancreatin, where factors such as stirring time significantly impacted the outcomes. The new protocol addresses this by specifying that stock solutions of all enzyme preparations should be stirred, in an ice bath, for 20 min and, once prepared, used within 30 min. Another important change was the substrate used; the previous **potato starch reference** (Sigma-Aldrich® 33,615) often resulted in turbid solutions suggesting incomplete solubilisation. Switching to a different potato starch (Sigma-Aldrich® S2004) and including an ethanol (80% v/v) wetting step eliminated this issue. The other two major changes concerned the **incubation** and **sample collection steps**. The original assay, based on a 3-min incubation at 20 °C with a single endpoint measurement posed a number of challenges. (A) Some laboratories reported difficulties attaining this temperature due to uncontrollable factors affecting ambient temperature (*e.g.* geographical location, timing of the experiments). (B) Additionally, the brief incubation meant that initial temperature differences in enzyme and/or substrate solutions and consequent delays reaching temperature equilibrium could significantly impact the results. (C) Lastly, the single sampling point, prevented examination of the reaction curve, making it impossible to confirm linearity. To address these elements of variability, the newly optimized protocol extends incubation time to 12 min and incorporates four sampling points (at 3, 6, 9 and 12 min). An example of a nonlinear reaction curve from an overly concentrated enzyme solution is presented in Figure S2 (Supplementary material) to illustrate the usefulness of the longer incubation in assessing experimental outcomes. This work also revealed practical experimental challenges. The importance of consistently performing good laboratory practices cannot be overstated – working within the weighing capacity and readability ranges of analytical scales, pipette checks and good pipetting practices are some examples of basic, routine tasks which can have significant repercussions on results. Further to these, we have also identified issues specific to this assay and compiled comprehensive troubleshooting advice in Table 2 to address these challenges. Finally, it is also important to note that the current protocol proposes a scaled down version of the assay from working volumes of 1.5 to 12 mL to 1 to 2 mL, adapted to a microplate format. This approach minimizes waste, in line with green chemistry principles^17^, and reduces the cost of the assay while facilitating parallel sample testing. This method of determining α-amylase activity through reducing sugar quantification remains simple, robust and fast. For mechanistic insight into the specific metabolites produced, other platforms, such as HPLC-based methods can be employed^18–20^.

**Table 2:** Troubleshooting advice.

Step	Problem	Possible Reason	Suggested solution
4.5.3 DNSA colour reagent	Precipitation	Low storage temperature	Heat the DNSA solution (maintaining the temperature ~ 45 °C) under constant stirring for 10–15 min
4.5.4 starch solution	Cloudy solution	Wrong potato starch reference	Make sure that the potato starch reference used is the same as that recommended in the text
		Starch not fully gelatinized	Make sure that the starch solution is thoroughly heated during preparation
4.4.5 α-amylase solutions	Enzyme preparation not properly dispersed	Solubility issues	Make sure that the solution is mixed homogenously for 20 min in an ice bath. Certain solutions (e.g. pancreatin tested here), can sometimes be cloudy. In the present study, the recommended dispersion step was effective as reflected by the similar repeatability and reproducibility as compared with the other references tested
			If poor solubility is a suspected cause of inconsistent results: Sonication may enhance solubility and subsequent centrifugation may reduce cloudiness^37^. However, depending on the pre-treatment followed, removal of insoluble material may reduce enzymatic activity by as much as 10%^38^. It is therefore recommended to investigate the impact of changing the enzyme preparation method by comparing results obtained when using different pre-treatments. Regardless of the method selected, the procedure followed when determining enzyme activity should be adopted for subsequent experiments
4.6 enzymatic assay	Inconsistent results and difficulty maintaining target temperature during incubations	Different instruments may require different temperature settings to ensure that the correct temperature is reached within the microtubes	Check the temperature reached within the microtubes:
			1- Set the water bath or heating block to 37 °C
			2- Place a microtube containing 1 mL of water in it
			3- Monitor internal temperature using a thermocouple until equilibrium is reached
			4- If the temperature inside the microtube is not 37 °C, adjust the settings as appropriate and continue monitoring until an internal temperature of 37 °C is reached and maintained stable over at least 15 min
		Substrate solution not at 37 °C	The substrate solution should be pre-heated to 37 °C to ensure that the temperature equilibrium is reached quickly
	High variability between repetitions	Inconsistent sampling times	It is important to be accurate to the secondSignificant (> 5 s) deviations from the sampling times and failing to immediately inactivate α-amylase straight after by dispensing the sample into a microtube pre-filled with DNSA are likely to have a significant impact on the results
	Inconsistent absorbance values	Dilution with water performed after boiling	The addition of water to the sample/calibrator and DNSA mixtures should only be carried out after these are heated to 100 °C for 15 min
	Flat or nonlinear reaction curve^1^	Enzyme solution too concentrated	Re-test the product at lower concentrations
	Absorbance close to zero and (almost) flat reaction curve	Enzyme solution too diluted	Re-test the product at higher concentrations

^1^Examples of curves obtained with solutions that were too concentrated are presented in Figure S2 in the Supplementary material for reference purposes.

Current INFOGEST protocols specify α-amylase activity levels based on measurements at 20 °C, according to Bernfeld’s original assay^12^. Therefore, it is very important that it remains possible to establish a relationship between the new protocol version described here (at 37 °C) and Bernfeld’s original procedure. In this context, a subgroup of five laboratories performed a parallel examination of all enzyme samples applying the new protocol at 20 °C enabling to determine the following conversion factor: one α-amylase unit, measured according to the protocol presented here (at 37 °C) equals 3.3 units at 20 °C. For future research, verifying unit definitions and making appropriate calculation adjustments when planning digestion studies is essential. When using the current, optimized protocol (at 37 °C) to characterise salivary and pancreatic α-amylases for in vitro digestions where recommended target activity levels are based on the original (20 °C) assay, the results should be converted to 20 °C-based units (*i.e.* divided by 3.3). This ensures correct reproduction of target α-amylase activity levels as recommended in the current INFOGEST protocols. It should be noted that this conversion factor has only been validated for the four products studied here and on the potato starch substrate used as part of the current study. For other enzyme preparations (and/or substrates), where a temperature conversion factor is required, it is recommended to perform the new assay at both temperatures.

In terms of assay performance, it is noteworthy that, although the number of laboratories carrying out the assay at 20 °C and 37 °C differed, repeatability (within-laboratory precision) improved for all porcine pancreatic samples at the higher temperature. In contrast, repeatability for the human saliva sample decreased, as indicated by an increase in CV_*r*_ from 9.7% at 20 °C to 12.5% at 37 °C. In parallel, assay reproducibility (between-laboratory agreement) improved for all samples at 37 °C. The contrasting trend observed for the saliva sample could be attributed to a combination of factors. First, differences in kinetic properties, substrate specificity, and catalytic efficiency between human salivary and porcine pancreatic α-amylases may play a role. Second, the human saliva sample was an unprocessed biological fluid, whereas the porcine pancreatic samples were processed extracts likely subjected to varying degrees of purification. The inherent complexity of saliva—as an aqueous matrix containing electrolytes (including bicarbonate), proteins (such as enzymes, immunoglobulins, glycoproteins, and albumin), mucins, glucose, urea, and ammonia^21^—brings about inevitable interactions between its components which can influence the activity and stability of α-amylase. Furthermore, temperature-led differences in sensitivity or susceptibility to these matrix interactions could influence salivary α-amylase’s behaviour differently at each temperature tested, and at 37 °C, where its activity is highest, such effects may be amplified. Nonetheless, the modest increase in CV_*r*_ remained within acceptable limits for most biochemical assays. If enhanced precision is required, further sample processing steps could be tested (such as using partially purified saliva or adding stabilizing agents for example).

It is also important to discuss the activity levels found in the products tested, particularly the human saliva and pancreatin, in relation to current recommendations for in vitro digestions. At present the INFOGEST research network consensus specifies target α-amylase concentrations of 75 and 200 U/mL respectively in the final oral and intestinal mixtures of static protocols to mimic adult digestions. In semi-dynamic studies, it is recommended to prepare the oral fluid at 150 U/mL and mix it with food at a 1:1 ratio (volume of SSF to dry weight of food)^11^. For the study of digestion in older adults, a 20% reduction in enzymatic activity is recommended during the intestinal phase, equating to 160 U/mL of α-amylase activity in the final digestion volume. All these figures are based on determinations of α-amylase activity at 20 °C. At the same temperature, we found amylolytic activities of 253.3 ± 52.1 U/mL in human saliva and 58.9 ± 5.5 U/mg in pancreatin. For the simulation of oral digestion, the target α-amylase activity in the INFOGEST static digestion protocol is integrated in a detailed procedure recommending that food is mixed with simulated oral fluid in a ratio of 1:1 (wt/wt)^9^. If the human saliva sample tested here was used in place of this simulated oral fluid, the α-amylase activity in the final bolus would be around 126.7 U per mL (or g) of bolus, *i.e.* 68.9% higher than the recommended activity levels for the oral phase. A similar challenge could be encountered during intestinal digestions, where the commercial pancreatin reference tested here is routinely used. This preparation contains different pancreatic enzymes. In addition to α-amylase, lipase (EC 3.1.13) and proteolytic enzymes, such as trypsin (EC 3.4.21.4) and chymotrypsin (EC 3.4.21.1), are also present. The INFOGEST protocol for replicating adult digestions in vitro, recommends that pancreatin concentrations are adjusted to obtain a trypsin activity of 100 U/mL in the final intestinal mixture (one trypsin unit being defined as the amount of enzyme that hydrolyses 1 μmol of p-toluene-sulfonyl-L-arginine methyl ester per minute at 25 °C and pH 8.1)^9,22^. In parallel, target α-amylase activity is 200 U/mL in the final intestinal digestion volume (as measured using the previous version of the protocol at 20 °C). This represents a 2 to 1 ratio between α-amylase and trypsin units during the intestinal digestion step. The pancreatin lot tested in the present study exhibited an amylolytic activity of 58.9 ± 5.5 U/mg at 20 °C (Table 1 – Panel A) which is within the range of values normally observed in our laboratories. Based on the reference method recommended in the INFOGEST consensus protocol^9^, typical trypsin activities for different lots of the same pancreatin reference, vary between 6 and 10 U/mg (unpublished data). Therefore, it seems that the typical concentrations in the porcine pancreatin most commonly used for in vitro digestions suggest actual ratios of α-amylase to trypsin ranging from 6:1 to 10:1 units, *i.e.* three to five fold above the ratio of the target activity levels. It is important to note that the results reported here reflect α-amylase activities from single batches of either saliva or pancreatin. While they are comparable to those often obtained in our laboratories, these results cannot be generalised. Nevertheless, our work highlights potential mismatches between recommended and actual α-amylase activity levels during in vitro digestions. This confronts us with an inevitable question: if similar observations are made when setting up digestion experiments according to INFOGEST protocols, should adjustments be made to align with current recommendations? Formulating the correct answer, however, is a challenging task which requires further investigation. The literature suggests that, to a certain extent, higher α-amylase levels or α-amylase to trypsin ratios may be physiologically accurate. Indeed, in Bernfeld’s own examinations of a centrifuged saliva sample, the amylolytic activity was about 1.6 fold higher than that of the pooled (non-centrifuged) sample tested here (410 U/mL)^12^. Using the same protocol, other researchers found intermediary activity levels in a commercial sample of pooled saliva (352 ± 41 U/mL)^23^. For intestinal digestions, comparisons between the activities of porcine and human pancreatic α-amylases on different starches (corn, rice, wheat and black bean) revealed that the human enzyme was generally more efficient, leading to higher degrees of hydrolysis (except for rice starch)^24^. Determinations of α-amylase activity in the human small intestine using Bernfeld’s method are scarce, but similar assays have been used. One study of 15 intestinal samples collected from three healthy men in the 2–3 h following the consumption of a liquid meal (containing vegetable fat, egg white and soluble starch) found mean α-amylase activity of 142 U/mL (range 50—250 U/mL) at 25 °C using pre-gelatinized starch as substrate^25^. A separate investigation of pancreatic enzyme secretion in 25 adults following stimulation with pancreozymin reported mean α-amylase output of 850 U/min (range 400 – 1780 U/min) and trypsin output of 136 U/min (range 55.5 to 335 U/min)^26^, yielding an α-amylase to trypsin output ratio of 6:1 units. In this study, α-amylase activity was determined using pre-gelatinized starch as substrate during a 10-min at 25°C^26,27^ incubation and the trypsin activity assay was similar to that in INFOGEST protocols (except for a shorter incubation, 3 *vs.* 10 min)^26,28^. Therefore, our results, combined with literature evidence, suggest a need to further investigate the relationships between α-amylase activities measured in vivo, current recommendations in digestion protocols, and possible implications for the outcomes of in vitro experiments.

## Methods

### Participating laboratories

Coordinating laboratory: Teagasc Food Research Centre, Moorepark, Fermoy, Co Cork P61 C996, Ireland.

Participating laboratories:

- Laboratory of Food Chemistry and Biochemistry, Department of Food Science and Technology, School of Agriculture, Aristotle University of Thessaloniki, P.O. Box 235, 54124, Thessaloniki, Greece
- Global Oatly Science and Innovation Centre, Rydbergs Torg 11, Space Building, Science Village, 22 484 Lund, Sweden
- Laboratory of Food Technology, Department of Microbial and Molecular Systems (M2S), KU Leuven, Kasteelpark Arenberg 23, PB 2457, 3001, Leuven, Belgium
- INRAE, Institut Agro, STLO, 35042 Rennes, France
- School of Biosciences, Faculty of Health and Medical Sciences, University of Surrey, Guildford, GU2 7XH, United Kingdom
- Nofima AS, Norwegian Institute of Food, Fisheries and Aquaculture Research, PB 210, N-1433, Ås, Norway
- Center for Innovative Food (CiFOOD), Department of Food Science, Aarhus University, Agro Food Park 48, Aarhus N 8200, Denmark
- Department of Horticulture, Martin-Gatton College of Agriculture, Food and Environment, University of Kentucky, Lexington, Kentucky, USA
- Department of Agricultural, Food, Environmental and Animal Sciences, University of Udine, Italy
- Wageningen Food & Biobased Research, Wageningen University & Research, 6708 WG Wageningen, The Netherlands
- Quadram Institute Bioscience, Rosalind Franklin Road, Norwich Research Park, Norwich, NR4 7UQ, United Kingdom
- Department of Food Engineering, Faculty of Engineering, Ege University, 35100, İzmir, Türkiye

### Materials

The chemicals and four test products used in the ring study are presented in (Table 3). They were ordered by the coordinating laboratory, aliquoted and shipped to each of the participating laboratories. All laboratories received aliquots from the same batch of each product, with the exception of 3,5-dinitrosalicylic acid (DNSA) which came from two different lots. Prior to shipping, calibration curves established with solutions prepared from both of these lots were compared, and showed nearly equivalent results (Figure S1 in Supplementary material-Section “Protocol implementation at each laboratory”).

**Table 3:** Products supplied to the laboratories participating in the ring trial.

Panel A: chemicals				
Name	CAS number	Purity	Product code	Brand
Sodium phosphate monobasic anhydrous	7558–80-7	≥ 99.0%	S0751-100G	Sigma-Aldrich
Sodium chloride	7647–14-5	≥ 99.5%	71,380-1 KG-M	Sigma-Aldrich
Sodium phosphate dibasic anhydrous	7558–79-4	≥ 99.0%	BP332-1	Fisher Chemical™
Starch from potato	9005–25-8	N/A	S2004-500G	Sigma-aldrich
3,5-dinitrosalicylic acid	609–99-4	≥ 98%	D-0550	Sigma-aldrich
Potassium sodium tartrate tetrahydrate	6381–59-5	≥ 99.0%	S2377-1 KG	Sigma-aldrich
Maltose monohydrate	6363–53-7	General purpose grade	M/1450/48	Fisher chemical™

Panel B: Test products				
Commercial name	Designation in the manuscript	Origin	Product code^1^	Brand
α-amylase (porcine pancreatic)	α-amylase M	Porcine pancreas	E-PANAA-12G	Megazyme
Pancreatic α-amylase from porcine pancreas Type VI-B	α-amylase S	Porcine pancreas	A3176-500KU	Sigma-aldrich
Pancreatin from porcine pancreas	Pancreatin	Porcine pancreas	P7545-25G	Sigma-aldrich
Saliva, pooled Human Donors	Human saliva	Pool from 10 healthy adults	991-05-P	Medix biochemica

^1^The batch numbers of the products tested can be found in the supplementary material.

### Equipment needed

The list of equipment required is provided as guidance below.

#### Preparation of reagents and enzyme solutions

Vortex mixer, pH meter with glass electrode, heating/stirring plate, incubator.

#### Enzyme assay

Water-bath or thermal shaker (*e.g.* PCMT Thermoshaker, Grant Instruments, United Kingdom) for enzyme–substrate incubations at 37 °C. Boiling bath (*e.g.* SBB Aqua 5 Plus, Grant Instruments, United Kingdom) or thermal shaker (*e.g.* PCMT Thermoshaker, Grant Instruments, United Kingdom) suitable for use at 100 °C. Spectrophotometer (*e.g.* Shimadzu UV-1800 Spectrophotometer, Shimadzu Corporation, Japan) or plate reader (*e.g.* BMG Labtech CLARIOstar Plus, BMG Labtech, Germany).

#### Basic materials

Volumetric flasks, heatproof bottle with lid (*e.g.* Duran bottle), magnetic stirrer, timer, thermocouple, safe lock microtubes (2 or 1.5 mL), heat (and water) resistant pen or labels for the microtubes, disposable standard cuvette or disposable polystyrene 96-well plate.

### Preparation of reagents and enzymes

#### 20 mM Sodium phosphate buffer (with 6.7 mM sodium chloride, pH 6.9 ± 0.3)

Prepare a stock solution by dissolving 1.22 g NaH_2_PO_4_ (anhydrous form), 1.38 g Na_2_HPO_4_ (anhydrous form) and 0.39 g NaCl in 90 mL purified water and make up the volume to 100 mL. Before use, dilute 10 mL of stock solution to 95 mL with purified water. Confirm that the pH of the buffer, when heated to 37 °C, is within the required working range (pH 6.9 ± 0.3). If needed, adjust the pH, using 1 M NaOH or HCl as required, before making up the volume to 100 mL.

#### Maltose calibrators

Prepare a 2% (w/v) maltose stock solution in phosphate buffer. Prepare a calibrator series by diluting the maltose stock solution in phosphate buffer as indicated in Table S2 (Supplementary Material – Section “Protocol implementation at each laboratory”). Store in the fridge (or freezer if not for use during the same day).

#### Colour reagent (96 mM DNSA with 1.06 M sodium potassium tartrate)

Dissolve 1.10 g of DNSA in 80 mL of 0.50 M NaOH at 70 °C in a glass beaker or bottle (partly covered to limit evaporation) on a pre-heated heat/stir plate with continuous stirring and temperature monitoring (*e.g.* using a thermocouple). Once the DNSA is fully dissolved, add 30 g of sodium potassium tartrate and continue stirring until it dissolves. Remove from heat and wait until the solution cools to room temperature. Bring to 100 mL with purified water. Store at room temperature protected from light for up to 6 months. If precipitation occurs during storage, re-heat to 45 °C while stirring on a heat-stir plate.

#### Starch solution

Potato starch pre-gelatinized in sodium phosphate buffer (1.0% w/v) is used as substrate. Pre-heat a heat-stir plate (setting it to 250 °C—300 °C is suggested) and pre-heat an incubator (or water bath) to 37 °C. Weigh 250 mg of potato starch into a heatproof bottle and add 750 μL of ethanol (80% v/v). Stir on a vortex mixer to wet all the starch powder (this is a critical step for the complete solubilisation of the starch). Add 20 mL of sodium phosphate buffer and mix again using a vortex mixer making sure that the powder is fully dispersed and there are no lumps in the solution. Cover the bottle with the lid to minimize evaporation (but making sure it is loose enough to let out excess steam) and place on the pre-heated heat-stir plate stirring at 180 rpm. When the solution starts bubbling, start the timer and boil on the heat-stir plate stirring continuously for exactly 15 min. Cool in the incubator/water bath for 15 min (or until it is safe to handle). Make up the volume of the starch solution to 25 mL in a volumetric flask by adding purified water. Store the solution in a closed bottle in an incubator (or water bath set to 37 °C) and use within 2 h. If the starch solution does not clarify significantly a new solution needs to be prepared, as this may indicate poor solubilisation and or gelatinization of the starch. Prepare a fresh solution each time as storing or freezing can cause starch retrogradation and influence the results of the assay.

#### α-amylase solutions

**Fig. 6:**
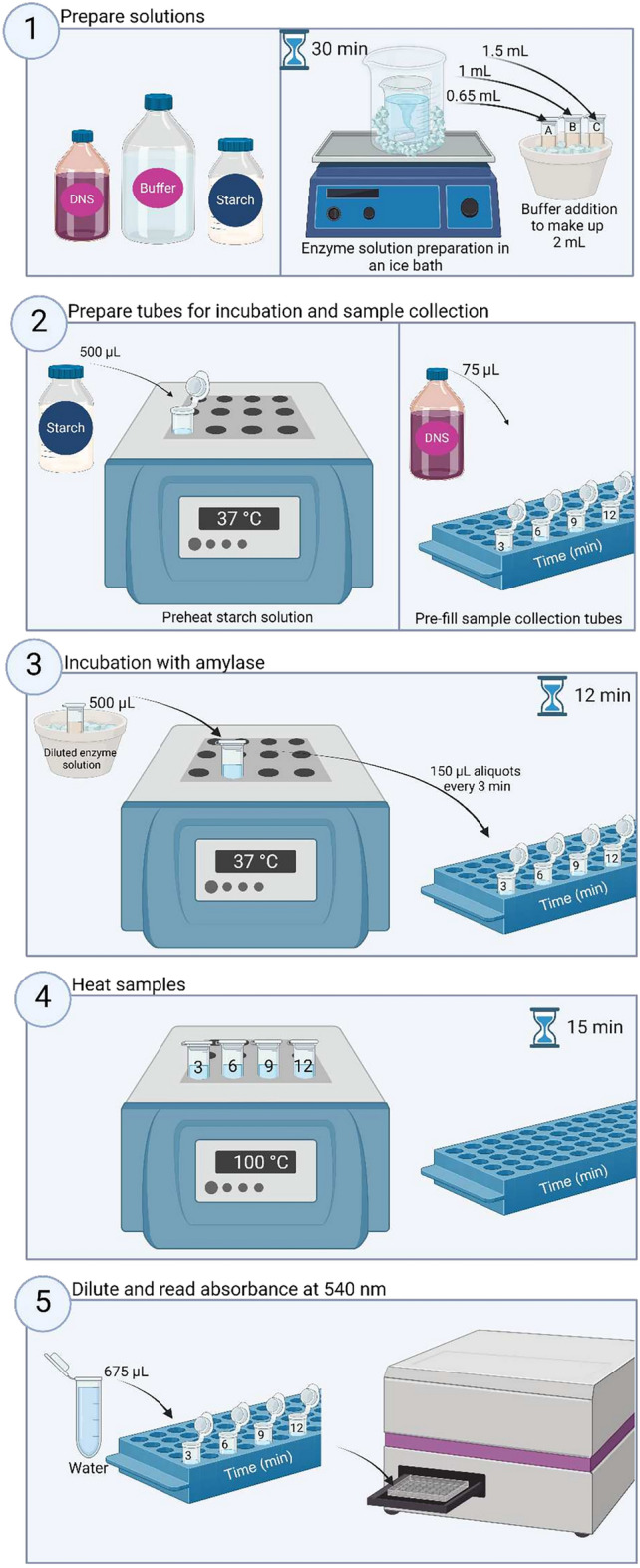
Schematic overview of the enzyme assay. Created in BioRender.com.

The preparation of the enzyme solutions is a critical step. Solutions prepared from enzyme powders should be carefully prepared following the same protocol each time to ensure adequate powder hydration and dispersion. After weighing the enzyme powder and adding the adequate amount of sodium phosphate buffer, stock solutions should be stirred in an ice bath (at around 250 rpm) for 20 min before any further dilutions (Graphical protocol in Fig. 6 and Picture S1 in the Supplementary Material). Subsequent dilution(s) of the stock solution(s) should be performed using sodium phosphate buffer to reach the recommended enzyme concentration of 1.0 ± 0.2 U/mL. For the four products tested in the ring trial, recommended concentrations are provided as reference in Table S7 (Supplementary material). For enzyme preparations, it is recommended to start from a stock solution prepared by adding 20 – 100 mg of enzyme powder to 25 mL of sodium phosphate buffer. For human saliva, a stock solution can be prepared by mixing 80 µL of saliva with 920 µL of buffer.

Each enzyme should be tested at three different concentrations prepared by diluting 0.65 mL, 1.00 and 1.50 mL of enzyme stock solution with 1.35, 1.00 and 0.50 mL of buffer, respectively (Table S3). These diluted enzyme solutions are referred to as solutions C1, C2 and C3. Enzyme solutions should always be kept on ice and used within 30 min of preparation.

### Enzymatic assay

An overview of the enzyme assay is presented in (Fig. 6).

#### Preparative procedures

Before starting, the following preparations are recommended: set the heating-block (water bath) as required to ensure 37 °C inside the microtubes (see troubleshooting advice, Table 2); pre-warm the starch solution to 37 °C; prepare a polystyrene container with ice.

#### Sample collection tubes

For each incubation that will be carried out, label and pre-fill four microtubes with 75 μL of DNSA colour reagent.

#### Incubations

Set three microtubes (one for each diluted enzyme solution C1, C2 and C3) in the preheated thermal shaker and let the temperature equilibrate before adding 500 µL of pre-warmed potato starch solution to each tube (maintain the tubes closed until the enzyme is added to prevent evaporation). Add 500 µL of diluted enzyme solution C1, C2 and C3 to the corresponding tubes at regular intervals. It is recommended to start the timer immediately when the α-amylase solution is added to the first tube and leave a 30 s interval before each subsequent addition.

#### Sample collection

Take a 150 μL aliquot from each tube after 3, 6, 9 and 12 min of incubation (respecting the order and intervals at which the incubations were initiated) and transfer it immediately to the corresponding sample collection tube pre-filled with DNSA to stop the reaction. Each aliquot should be taken as closely as possible to its respective sample collection time, within a maximum of ± 5 s.

#### Absorbance measurements

Prepare the maltose calibrators by mixing 150 µL of each maltose calibrator with 75 µL of DNSA reagent. Centrifuge the samples and calibrators (1000 g, 2 min) so that all droplets are brought back into solution. Place the samples and calibrators in the thermal shaker (or boiling bath) (100 °C, 15 min) and then transfer them to an icebox to cool for 15 min. Add 675 µL of purified water to each tube and mix by inversion. Transfer the samples and calibrators to a cuvette or pipette to a microtiter plate (300 µL per well) and record the absorbance at 540 nm (A_540nm_).

### Ring trial organization

#### Preliminary testing

Throughout the protocol optimization phase, the assay was repeated multiple times by the coordinating laboratory to define practical aspects. Each of the four test products has been assayed at different concentrations. The final test concentrations were defined by choosing a test concentration that allowed for an adequate distribution of the endpoint measure (spectrophotometry absorbance) and communicated to the participating laboratories.

#### Protocol transference

A detailed written protocol (Supplementary material) was transferred to each participating laboratory including the recommendations for concentrations of the test products. All laboratories were invited to an online training session that included a video of the assay followed by a Q&A session to clarify any doubts. All labs carried out the assay and reported their results on a standard Excel file between May and November 2023.

#### Incubation temperatures

All laboratories tested the four enzyme preparations at 37 °C as described above. A subgroup of five laboratories also repeated the assays at 20 °C with the purpose of trying to establish a correlation between the results obtained at both temperatures.

For incubations at 20 °C protocol adaptations were performed as follows. A different recipe was used to prepare the 200 mM sodium phosphate buffer stock solution. It consisted of 1.26 g NaH_2_PO_4_, 1.29 g Na_2_HPO_4_ and 0.39 g NaCl. The dilutions (10 mL stock diluted to 95 mL with purified water) and pH (6.9 ± 0.3) were the same as those for the buffer used at 37 °C. All reagents and solutions requiring the use of buffer were freshly prepared using this buffer recipe. The recommended concentrations of the α-amylase stock solutions were adjusted to ensure that enough enzymatic activity was present.

### Calculations

#### Calibration curve

The A_540nm_ of the colour reagent blank was subtracted from the readings of all maltose calibrators and their concentration (mg/mL) was plotted against the corresponding ΔA_540nm_. For reference purposes, using a 96 well plate, the absorbance at 540 nm should increase linearly from approximately 0.05 (for the colour reagent blank) to 1.5 for the highest maltose concentration. The calibration blank should not be included as a data point in the calibration curve.

#### Enzyme activity definition

The definition of α-amylase activity resulting from the application of the newly developed protocol is the following:

- Based on the definition originally proposed by Bernfeld: one unit liberates 1.0 mg of maltose equivalents from potato starch in 3 min at pH 6.9 at 37 °C.
- Based on the international enzyme unit definition standards: one unit liberates 1.0 μmol of maltose equivalents from potato starch in 1 min at pH 6.9 at 37 °C.

Amylase activity units based on the definition originally proposed by Bernfeld were multiplied by the conversion factor 0.97 to convert the result into IU.

#### Enzyme activity calculation

The first step was to subtract A_540nm_ of the colour reagent blank from all readings. The calibration curve was then used to calculate the maltose concentrations (mg/mL) reached with each diluted enzyme solution (C1, C2 and C3) at each sampling point during incubations. Enzyme concentrations during incubations were then calculated as mg/mL for enzyme powders, or µL/mL for liquid (saliva) samples.

For each diluted enzyme solution (C1, C2 or C3), maltose concentrations (mg/mL) were plotted against time (*t*_min_) and the corresponding linear regression was established to determine the reaction kinetics’ slope ($\text{m}t{\text{min}}$). For each enzyme concentration, units of enzyme were calculated using the following equation.

$$Activity (U per mg or \mu L of enzyme product)= 3min\times \frac{\text{m}t{\text{min}}(\frac{maltose concentration (\frac{mg}{mL})}{time (min)})}{Enzyme concentration \left(\frac{mg}{mL} or\frac{\mu L}{mL}\right)}$$

A template Excel file is provided for calculations in the Supplementary Material.

### Statistical analysis and assessment of method’s performance

Data visualization and statistical analyses have been performed in R (version 4.3.2)^29^. The packages ggplot2^30^ and ggdist^31^ have been used in the preparation of the plots presented in the manuscript.

Outlier analysis was conducted on non-transformed data to preserve the original variability and scale of the datasets. First, Cochran’s test (outliers package in R^32^) was used to assess intralaboratory variability and did not reveal any outliers. Subsequently, for interlaboratory comparisons, boxplot analysis, Bias Z-scores and Grubbs’ test^32^ were employed complementarily. The results reported by one lab for three test products (pancreatin, α-amylase M and α-amylase S) assayed at 37 °C were more than 1.5 interquartile ranges below the 25^th^ or above the 75^th^ percentiles, consistent with unsatisfactory Bias Z-scores (|z|> 3). Grubb’s test confirmed these as outliers and they have been excluded from the statistical analysis. All results in the 20 °C dataset fell within 1.5 interquartile ranges of the 25^th^ and 75^th^ percentiles (Fig. 5), consistent with satisfactory Bias Z-scores (|z|< 2) (Supplementary Figure S4). While Grubbs’ test identified two potential outliers (Lab A for pancreatin and Lab D for α-amylase M), this outcome was considered less reliable due to the small sample size (n = 5) and lack of corroboration from boxplot and Bias Z-score analyses, and so these results were retained.

Statistical analysis of the dataset resulting from the implementation of the protocol at 37 °C has been carried out to investigate the effects of the tested products, concentrations and incubation conditions (thermal shaker *vs.* water bath with or without shaking) as well as the two-way and three-way interactions between these factors. Normality of this dataset has been confirmed through the Shapiro–Wilk test (p > 0.05). The homogeneity of variances, as assessed using Levene’s test in the Rstatix package version 0.7.2^33^, was not confirmed (p < 0.001). Due to the limited availability of suitable non-parametric alternatives, a logarithm transformation was performed on this data set enabling homogenisation of the variances and application of a three-way ANOVA (Rstatix package). Statistically significant effects were further examined using Pairwise T-Test comparisons, applying Bonferroni adjustments for multiple comparisons as required. The results obtained when implementing the protocol at 20 °C were normally distributed, but homogeneity of variances was not confirmed for this dataset either. The corresponding logarithm transformed data frame did not conform to normality, hence the Kruskal–Wallis test was applied to examine the significance of the differences between the four products, followed by the Bonferroni-corrected Wilcoxon test for pairwise comparisons (all tests performed using the Rstatix package). Statistically significant effects have been accepted at the 95% level.

For each laboratory and product, an individual ratio of α-amylase activity at 37 °C to 20 °C was calculated, and the mean of these ratios across all laboratories was determined for each product. The 95% confidence interval for this mean ratio was computed using the t-distribution. Normal distribution and homogeneity of variances have been confirmed for this dataset, hence one-way ANOVA was used to investigate whether the ratios obtained for each product were significantly different.

For a thorough understanding of the method’s reliability, precision, and transferability across different laboratory settings three complementary metrics have been used: Z-scores based on bias scores for a standardized evaluation of systematic errors, repeatability and reproducibility.

Z-scores were calculated to standardize the comparison of bias scores across laboratories and products enabling to assess the overall agreement between individual laboratory results and the mean for each product. For each product, bias scores were first calculated for each laboratory using the mean of all laboratories as the reference value and then converted to z-scores:

$$\text{z }=\frac{\left( x -\text{ X}\right)}{\text{SD}}$$

x is the individual laboratory result, X is the mean of all laboratories, and SD is the standard deviation. Z-scores interpretation followed standard criteria with |z|≤ 2 as satisfactory, 2 <|z|< 3 as questionable, and |z|≥ 3 as potentially unsatisfactory.

Repeatability (measured as intralaboratory coefficient of variation, CV_r_), which quantifies method precision within each laboratory, reflecting consistency under identical conditions, was calculated as the root mean square of the individual laboratory’s CVs:

$${CV}_{r}=\sqrt{\frac{1}{L}\sum_{i=1}^{L}{\left({CV}_{i}\right)}^{2}}$$

CV_r_ is the coefficient of variation under repeatability conditions (intralaboratory); $i$ indexes each laboratory, ${CV}_{i}$ is the coefficient of variation for laboratory $i$; L is the number of participating laboratories.

Reproducibility (measured as coefficient of variation, CV_R_), a measurement of method’s consistency across different laboratories indicates its robustness to varying environments and operators, was calculated for each tested product as:

$${CV}_{R}=\frac{SD}{X} \times 100$$

CV_R_ is the coefficient of variation under reproducibility conditions (interlaboratory); SD and X correspond to the standard deviation and mean values calculated from interlaboratory data.

Coefficients of variation below 30%^15,16^ are frequently considered to be indicators of small intra- and interlaboratory variability. In some cases, critical thresholds for repeatability (intralaboratory CV) are set at 20%^34^.

## Conclusion

The present interlaboratory study generated highly satisfactory results in terms of the feasibility, repeatability, and reproducibility of the proposed protocol to measure α-amylase activity. Based on the outcomes of this research, the following recommendations are now put forward with the aim of supporting the ongoing development of research in this field.

1. The newly optimized protocol validated through the present study should be adopted for future α-amylase activity determinations (replacing the earlier version, originally described by Bernfeld^12^) in conjunction with INFOGEST digestion protocols. An Excel® spreadsheet is provided in the supplementary material to facilitate calculations.
2. One α-amylase activity unit measured at 37°C, as described in the present study, is equivalent to 3.3 units measured at 20°C. This conversion factor can be applied to the products studied here, where it is necessary to establish a correlation with activity levels based on the unit definition originally proposed by Bernfeld (20°C based). This applies to the currently available INFOGEST static^9^ and semi-dynamic^11^ models of adult digestion and related adaptations for population groups differing according to factors such as sex^35^ or age, e.g. infants^36^ and older adults^10^. The Excel® spreadsheet provided in the supplementary material can also be used to work out conversions and correct concentrations when using the enzyme preparations tested in the present study. This conversion factor may not be applicable to other products. Therefore, it is recommended that different products are analysed on a case-by-case basis and, if a similar conversion factor is required, are assayed at both temperatures following an equivalent approach to that of the present study.
3. In future research focusing on starch digestion, the α-amylase activity(ies) in the preparation(s) used in oral and/or intestinal digestion fluids should be routinely measured and reported to ensure adequate result interpretation and facilitate comparisons across different studies.
4. Further work should be carried out to investigate how the recommended levels of α-amylase activity in *in vitro* digestion protocols compare with *in vivo* findings and the implications for protocol implementation and result interpretation. This will be crucial to evaluate the need (and provide directions) for further protocol optimization.

## Supplementary Information

Supplementary Information 1.

Supplementary Information 2.

## Data Availability

The data supporting the findings of this study is provided within the manuscript or supplementary information files. Should any raw data files be needed, they are available from the corresponding author upon reasonable request.
