# Supplementary material for: Interlaboratory validation of an optimized protocol for measuring α-amylase activity by the INFOGEST international research network
Source: Sci Rep. 2025 Aug 23;15:30985. doi: 10.1038/s41598-025-12561-y (PMC12373966; doi:10.1038/s41598-025-12561-y)
Supplement: Supplementary file 2 — Supplementary Information 2. [file 41598_2025_12561_MOESM2_ESM.docx]

# Interlaboratory validation of a protocol for measuring amylase activity in biological samples - Supplementary material

Daniela Freitas[[1]](#footnote-2)*, Shannon Gwala^1^, Gwénaële Henry[[2]](#footnote-3), Athina Lazaridou[[3]](#footnote-4), Christine Boesch[[4]](#footnote-5), Dorine Duijsens[[5]](#footnote-6), Faye Wheller[[6]](#footnote-7), Ivan M. Lopez-Rodulfo[[7]](#footnote-8), Kali Kotsiou^3^, Kendall R. Corbin[[8]](#footnote-9), Marilisa Alongi[[9]](#footnote-10), Mario M. Martinez^7^,[[10]](#footnote-11), Maryam S. Hafiz[[11]](#footnote-12), Monic M.M. Tomassen[[12]](#footnote-13), Natalia Perez-Moral[[13]](#footnote-14), Natalia P. Vidal^7^, Renata M.C. Ariëns^13^, Sebnem Simsek[[14]](#footnote-15), Sedef Nehir El^15^, Sibel Karakaya^15^, Steven Le Feunteun^2^, Shanna Bastiaan-Net^13^, Svenja Krause^17^, Bin Zhang[[15]](#footnote-16), Caroline Orfila[[16]](#footnote-17),4, Simon Ballance[[17]](#footnote-18)a, Terri Grassby^6a^

*Corresponding author [daniela.freitas@teagasc.ie](mailto:daniela.freitas@teagasc.ie)

^a^These authors contributed equally

# Table of Contents

[1. Protocol shared with the laboratories participating in the Interlaboratory study 3](#_Toc180076389)

[2. Results obtained during preliminary tests 20](#_Toc180076390)

[2.1. Comparison of the two DNSA lots shipped to the participating laboratories 20](#_Toc180076391)

[2.2. Examples of nonlinear reaction curves 21](#_Toc180076392)

[3. Results obtained during the ring trial 23](#_Toc180076393)

[3.1. Implementation of the protocol by the participating laboratories 23](#_Toc180076394)

[3.2. Calibration curves 25](#_Toc180076395)

[3.3. Repeatability (CV_r_) 26](#_Toc180076396)

# Detailed Protocol (adapted from the protocol shared with the laboratories participating in the Interlaboratory study)

**Principle:** Determination of reducing sugars released from starch (calculated as maltose equivalents) as measured by the reduction of 3,5-dinitrosalicylic acid (DNSA) detected by spectrophotometry at 540 nm.

**Unit definition:**

- Based on the definition originally proposed by Bernfeld: one unit liberates 1.0 mg of maltose equivalents from potato starch in 3 minutes at pH 6.9 at 37°C.

- Based on the international enzyme unit (IU) definition standards: one unit liberates 1.0 μmol of maltose equivalents from potato starch in 1 minute at pH 6.9 at 37°C.

Conversions between these two unit definitions can be made as follows:

1 Bernfeld unit = 0.97 IU.

**Conditions**: T = 37°C, pH = 6.9

**The protocol is adapted from:** P. Bernfeld, in Methods Enzymol., Academic Press, 1955, vol. 1, pp. 149–158.

**Key modifications to the original protocol** include the testing of the enzymatic reaction temperature at 37°C. This is more reflective of physiological conditions and it is also a more controllable temperature for most labs. Previously this assay was conducted at 20°C and therefore reference amylase units in INFOGEST protocols currently available refer to the assay conducted at 20°C. Five labs will be testing the same enzymes at both 20°C and 37°C to try to determine a conversion factor as this will be important to facilitate future work based on these protocols.

**Chemicals, equipment and materials**

**Chemicals and enzymes:**

- NaH_2_PO_4_ (Sigma-Aldrich S0751, MW = 119.98 g/mol)[[18]](#footnote-19)
- Na_2_HPO_4_ (Ficher Chemical^TM^ BP332-1, MW = 141.96 g/mol)^1^
- NaCl (Merck 71380-1KG-M)
- Soluble potato starch (Sigma-Aldrich S2004) – moisture content needs to be measured in advance[[19]](#footnote-20)
- 3,5-dinitrosalicylic acid (DNSA, Sigma-Aldrich D-0550)
- Potassium sodium tartrate tetrahydate (Sigma-Aldrich S2377)
- NaOH (Merck 221465)
- Pancreatic α-amylase (megazyme E-PANAA-4G)
- Pancreatic α-amylase (A3176, Sigma-Aldrich)
- Porcine pancreatin (P7545, Sigma-Aldrich)
- Human saliva (Lee Biosolutions, 991-05-P)
- D-Maltose monohydrate (Merck 5912[)](http://www.sigmaaldrich.com/ProductLookup.html?ProdNo=M5885&Brand=SIAL)
- Ultrapure type I water, generated by a Milli-Q system (referred in text as purified water)

**Equipment:**

- pH Meter with glass electrode
- Heating/stirring plate to make up the reagents and buffers
- Vortex mixer **>> Only used for the preparation of starch solution, calibrators and enzyme solutions. Vortexing should be avoided during the enzyme assay and mixing should only be performed by repetitive pipetting.**
- Microfuge or Eppendorf centrifuge
- Incubator set to the temperature of the assay, 20°C or 37°C (to cool the starch solution)
- Heating block set at 20°C or 37°C for enzyme digestion.
- Heating block (or boiling bath) that can go up to 100°C
- Spectrophotometer or plate reader. The readings will vary between equipment, please use the same equipment every time if possible and record this on the result sheet

**Materials:**

- Disposable standard cuvette (Light path = 1 cm), or 96 well plates (disposable polystyrene plates such as this example <https://www.sarstedt.com/en/products/laboratory/general-laboratory-products/micro-test-plates/product/82.1581.001/> work well)
- Safe lock Eppendorf tubes (2 or 1.5 mL)
- Calibrated micropipette (e.g. Gilson P200 and P1000) and tips, or positive displacement pipette.
- Volumetric flasks for solutions
- Glass beakers (with lid) or Duran bottle (with lid), glass stirring rod, magnetic stirrer
- Ice
- Heat and water resistant pen or labels for the tubes
- Timer
- Thermocouple

**Main protocol steps and guide to plan the experimental work**

| **Table S1.** – Main protocol steps | | | |
| --- | --- | --- | --- |
| **Procedures** | **Can be carried out in advance** | **Needs to be carried out on the same day as the enzyme assay** | **Page** |
| **I. Preparation of reagents and enzymes** | | | |
| 1. Sodium phosphate buffer |  |  | 6 |
| 2. Maltose |  |  | 7 |
| 3. DNSA Colour Reagent |  |  | 8 |
| 4 Starch solution |  | *To be used within 2 hours* | 8 |
| 5. α-Amylase solutions |  | *To be used within 30 min* | 9 |
| **II. Preparative procedures** | | | |
| 1. Thermo-shaker temperature checks |  |  | 13 |
| 2. Calibration curve to quantify the liberated maltose |  |  | 13 |
| 3. Moisture content of the starch |  |  | 13 |
| **III. α-Amylase assay procedure** | | | |
| 1. Preparation of the α-Amylase assay procedure |  |  | 14 |
| 2. Prepare the incubation tubes |  |  | 14 |
| 3. Prepare the sample collection tubes |  |  | 14 |
| 4. Prepare the three enzyme dilutions |  |  | 15 |
| 5. Start the reactions (6 Time-critical steps) |  |  | 15 |
| 6. Collect samples and stop the enzymatic reaction (6 Time-critical steps) |  |  | 15 |
| 7. Prepare the samples for the absorbance measurements |  |  | 15 |
| *Consider labelling all tubes in advance to save time on the day of the enzyme assay | | | |

**I. Preparation of reagents and enzymes**

1. **Sodium phosphate buffer**

Buffer: 20 mM sodium phosphate buffer with 6.7 mM sodium chloride, pH 6.9 ± 0.3.

The preparation will be slightly different depending on the temperature of use, either 20°C or 37°C**.**

**Buffer to use at 37°C**

*A recipe for a stock solution is provided. It is recommended that this is divided into 10x 10-mL aliquots which can be frozen and later thawed and diluted as needed for the experiments. Alternatively, if you plan to use up all the buffer within a few days, the diluted solution can be prepared by directly diluting the 100 mL of stock solution to 1 L.*

Stock solution to dilute before use:

To make a 200 mM phosphate stock solution, dissolve **1.2238 g** NaH_2_PO_4_, **1.3770 g** Na_2_HPO_4_ and **0.3913 g** NaCl in 90 mL purified water and make up the volume to 100 mL.

Before use, dilute 10 mL of stock solution to 95 mL with purified water. Confirm that the pH of the buffer, when heated to 37°C, is within the required working range (pH 6.9 ± 0.3). If needed, adjust with 1 M NaOH or HCl before making up the volume to 100 mL.

**Labs testing two incubation temperatures:**

**Buffer to use at 20°C**

*A recipe for a stock solution is provided. It is recommended that this is divided into 10x 10-mL aliquots which can be frozen and later thawed and diluted as needed for the experiments. Alternatively, if you plan to use up all the buffer within a few days, the diluted solution can be prepared by directly diluting the 100 mL of stock solution to 1 L.*

Stock solution to dilute before use:

To make a 200 mM phosphate stock solution, weight **1.2579 g** NaH_2_PO_4_, **1.2918 g** Na_2_HPO_4_ and **0.3913 g** NaCl in 90 mL purified water and make up the volume to 100 mL.

Before use, dilute 10 mL of stock solution to 95 mL with purified water. Confirm that the pH of the buffer, measured at 20°C, is within the required working range (pH 6.9 ± 0.3). If needed, adjust with 1 M NaOH or HCl before making up the volume to 100 mL.

1. **Maltose calibrators**

The maltose calibrators are prepared in sodium phosphate buffer. The buffer should be the same as the one used for the preparation of the enzyme and starch solutions.

**Labs testing two incubation temperatures:**

Please prepare a different calibrator series for each incubation temperature using the corresponding buffer.

- 1. The maltose stock solution shipped for the ring trial contains maltose in sodium phosphate buffer at a concentration of 2% (w/v).
  2. Prepare a calibrator series by diluting this stock solution (2% w/v) in buffer in 2 mL Eppendorf tubes as indicated in Table S2.
  3. Store in the fridge (or freezer if not using in the same day).

| **Table S2**: Preparation of maltose calibrators for calibration curve | | | | |
| --- | --- | --- | --- | --- |
| **Calibrator number** | **Volume of maltose stock (2%, w/v)**  **(µL)** | **Volume of buffer pH 6.9**  **(µL)** | **Maltose concentration**  **(mg/mL)** | **Absorbance at 540_nm_** |
| **0 (Colour reagent blank)** | 0 | 2000 | 0.0 | Results of preliminary tests using the same maltose stock solution shipped to all labs: absorbance at 540_nm_ increased linearly from 0.05 (colour reagent blank) to 1.5 (highest maltose concentration). |
| **1** | 20 | 1980 | 0.2 |
| **2** | 40 | 1960 | 0.4 |
| **3** | 60 | 1940 | 0.6 |
| **4** | 80 | 1920 | 0.8 |
| **5** | 100 | 1900 | 1.0 |
| **6** | 150 | 1850 | 1.5 |
| **7** | 200 | 1800 | 2.0 |
| **8*** | 250 | 1750 | 2.5 |
| **9*** | 300 | 1700 | 3.0 |
| *These concentrations have been added to the calibration curve to enable confirmation of linearity for a broader concentration range in case maltose concentration at the last time point of an incubation curve is > 2 mg/mL. | | | | |

1. **DNSA Colour Reagent**

The colour reagent is a solution of 96 mM 3,5-dinitrosalicylic acid (DNSA) with 1.06 M sodium potassium tartrate.

- 1. Slowly dissolve 1.1 g of DNSA (MW=228.1 g/mol) in 80 mL of 0.5 M NaOH at 70ºC in glass beaker on a pre-heated heat/stir plate.
  2. Monitor the temperature of the solution using a thermocouple and adjust the temperature settings of the heat/stir plate as needed.
  3. Add 30 g of sodium potassium tartrate (MW=282.2 g/mol) and stir until it dissolves.
  4. Remove from heat and wait until it cools to room temperature.
  5. Bring to 100 mL with purified water.
  6. Store DNSA reagent at room temperature protected from light (use amber bottles or cover the container with aluminium foil) for up to 6 months.
  7. If precipitation occurs during storage, re-heat the solution to 45°C while stirring on a heat-stir plate.

1. **Starch solution**

Potato starch pre-gelatinized in sodium phosphate buffer (1.0% w/v) is used as substrate. Prepare a fresh solution each time as storing or freezing will retrograde the starch and reduce its digestibility by amylase.

- 1. Pre-heat a heat-stir plate to 250-300°C.
  2. Pre-heat an incubator (or water-bath) to the temperature of the assay to be carried out (20°C or 37°C)
  3. Weigh 250 mg of potato starch into a heatproof bottle (such as a Duran bottle with lid) and add 750 μL of ethanol (80% v/v). Stir on a vortex mixer to wet all the starch powder. This is a critical step for the complete solubilisation of starch.
  4. Add 20 mL of sodium phosphate buffer (to use at 20°C or 37°C depending on the assay to be carried out) and mix again in a vortex mixer (make sure there are no lumps in your solution).
  5. Add a magnetic stirrer and loosely close the tube/bottle lid, to avoid water evaporation.
  6. Place on the pre-heated heat-stir plate and set the stirring rate to180 rpm. When the start solution starts bubbling, start the timer and let the solution boil on the heat-stir plate for exactly 15 minutes. Make sure that the lid is loose enough to let out excess steam.
  7. Let cool in the incubator/water bath for 15 minutes (or until it is safe to handle).
  8. Make up the volume of the starch solution to 25 mL in a graduated flask by adding purified water to make a final concentration of 1.0% (w/v) and mix well.
  9. Maintain the solution in the incubator/water bath set to the temperature of your assay and use within 2 hours.

1. ***
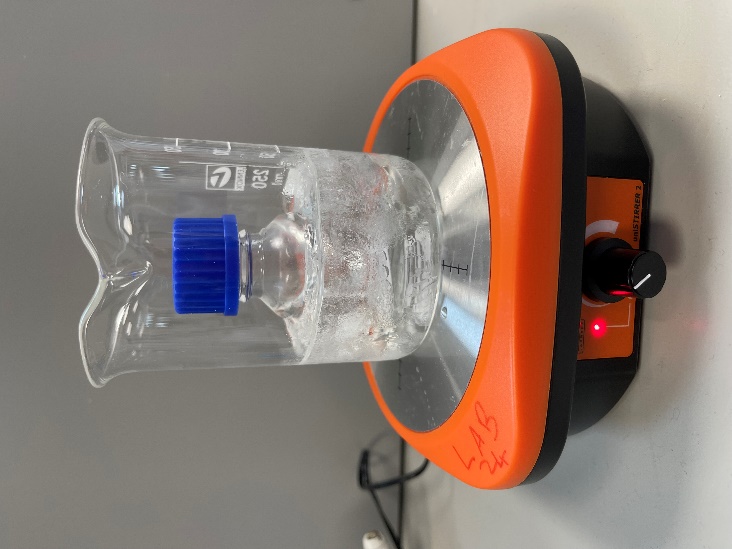
*α-Amylase solutions (Critical step)**

**Picture S1** - Ice-bath for the preparation of stock solutions from enzyme powders

**The preparation of the enzyme solutions is a critical step.**

Solutions prepared from enzyme powders should be carefully prepared following the same protocol each time to ensure adequate powder hydration and dispersion in the buffer. After weighing the enzyme powder and adding the adequate amount of buffer, please make sure that stock solutions are stirred in an ice bath (around 250 rpm) for 20 minutes before any further dilutions (Picture S1)

Keep enzyme solutions on ice at all times.

Use within 30 min of preparation.

All enzymes have been tested at Teagasc, before shipping, at both 20°C and 37°C.

**The recommended concentrations for incubations at 37°C, for each product, are presented below.**

- 1. Pancreatic amylase from Sigma Aldrich (A3176) (Lot number: **SLCP6815**) recommended dilutions for incubations at 37°C
- Dissolve 100 mg of the enzyme powder in 25 mL sodium phosphate buffer. Record the exact mass of enzyme weighed. Solubilise in an ice bath with continuous stirring (250 rpm) on a stirring plate for 20 minutes.
- Vortex the suspension and prepare a first dilution by transferring 2 mL into a falcon tube containing 8 mL of buffer.
- Vortex the solution and prepare a second dilution by collecting a 0.5 mL aliquot and adding to another falcon tube with 9.5 mL of buffer. Keep this solution on ice until you are ready to prepare the three enzyme concentrations for the incubations as described in Table S3.
- Do not let it sediment, if it does sediment, vortex again. Use within 30 min.
  1. Pancreatin from Sigma Aldrich (P7545) (Lot number: **SLCM8903**) recommended dilutions for incubations at 37°C
- Dissolve 20 mg of the enzyme powder in 25 mL sodium phosphate buffer. Record the exact mass of enzyme weighed. Solubilise in an ice bath with continuous stirring (250 rpm) on a stirring plate for 20 minutes.
- Vortex the suspension and prepare a first dilution by transferring 1 mL into a falcon tube containing 9 mL of buffer.
- Vortex the solution and prepare a second dilution by collecting a 0.5 mL aliquot and adding to another falcon tube with 9.5 mL of buffer. Keep this solution on ice until you are ready to prepare the three enzyme concentrations for the incubations as described in Table S3.
- Do not let it sediment, if it does sediment, vortex again. Use within 30 min.
  1. Pancreatic amylase from Megazyme (E-PANAA-4G) (Lot number: **201101C**) recommended dilutions for incubations at 37°C
- Dissolve 20 mg of the enzyme powder in 25 mL sodium phosphate buffer. Record the exact mass of enzyme weighed. Solubilise in an ice bath with continuous stirring (250 rpm) on a stirring plate for 20 minutes.
- Vortex the suspension and prepare a first dilution by transferring 750 µL into a falcon tube containing 9.25 mL of buffer.
- Vortex the solution and prepare a second dilution by collecting a 450 µL aliquot and adding to another falcon tube with 9.55 mL of buffer. Keep this solution on ice until you are ready to prepare the three enzyme concentrations for the incubations as described in Table S3.
- Do not let it sediment, if it does sediment, vortex again. Use within 30 min.
  1. Saliva from Lee Biosolutions (Lot number: **04J9237**) recommended dilutions for incubations at 37°C
- Let thaw in the fridge (4°C). There is a very small volume in the vials so it will thaw quickly.
- Vortex the vial and transfer 80 µL of saliva into an eppendorf containing 920 µL of buffer.
- Vortex and prepare a second dilution by transferring 150 µL of this solution into a falcon tube containing 9.85 mL of buffer. Keep this solution on ice until you are ready to prepare the three enzyme concentrations for the incubations as described in Table S3.
- Vortex again immediately before use. Use within 30 min.

**Additional information for labs testing two incubation temperatures:**

**Recommended concentrations for incubations at 20°C, for each product, are presented below.**

- 1. Pancreatic amylase from Sigma Aldrich (A3176) (Lot number: **SLCP6815**) recommended dilutions for incubations at 20°C
- Dissolve 200 mg of the enzyme powder in 25 mL sodium phosphate buffer. Record the exact mass of enzyme weighed. Solubilise in an ice bath with continuous stirring (250 rpm) on a stirring plate for 20 minutes.
- Vortex the suspension and prepare a first dilution by transferring 3 mL into a falcon tube containing 7 mL of buffer.
- Vortex the solution and prepare a second dilution by collecting a 0.5 mL aliquot and adding to another falcon tube with 9.5 mL of buffer. Keep this solution on ice until you are ready to prepare the three enzyme concentrations for the incubations as described in Table S3.
- Do not let it sediment, if it does sediment, vortex again. Use within 30 min.
  1. Pancreatin from Sigma Aldrich (P7545) (Lot number: **SLCM8903**) recommended dilutions for incubations at 20°C
- Dissolve 80 mg of the enzyme powder in 25 mL sodium phosphate buffer. Record the exact mass of enzyme weighed. Solubilise in an ice bath with continuous stirring (250 rpm) on a stirring plate for 20 minutes.
- Vortex the suspension and prepare a first dilution by transferring 1 mL into a falcon tube containing 9 mL of buffer.
- Vortex the solution and prepare a second dilution by collecting a 0.5 mL aliquot and adding to another falcon tube with 9.5 mL of buffer. Keep this solution on ice until you are ready to prepare the three enzyme concentrations for the incubations as described in Table S3.
- Do not let it sediment, if it does sediment, vortex again. Use within 30 min.
  1. Pancreatic amylase from Megazyme (E-PANAA-4G) (Lot number: **201101C**) recommended dilutions for incubations at 20°C
- Dissolve 25 mg of the enzyme powder in 25 mL sodium phosphate buffer. Record the exact mass of enzyme weighed. Solubilise in an ice bath with continuous stirring (250 rpm) on a stirring plate for 20 minutes.
- Vortex the suspension and prepare a first dilution by transferring 1 mL into a falcon tube containing 9 mL of buffer.
- Vortex the solution and prepare a second dilution by collecting a 0.5 mL aliquot and adding to another falcon tube with 9.5 mL of buffer. Keep this solution on ice until you are ready to prepare the three enzyme concentrations for the incubations as described in Table S3.
- Do not let it sediment, if it does sediment, vortex again. Use within 30 min.
  1. Saliva from Lee Biosolutions (Lot number: **04J9237**) recommended dilutions for incubations at 20°C
- Let thaw in the fridge (4°C). There is a very small volume in the vials so it will thaw quickly.
- Vortex the vial and transfer 80 µL of saliva into an Eppendorf containing 920 µL of buffer.
- Vortex and prepare a second dilution by transferring 400 µL of this solution into a falcon tube containing 9.6 mL of buffer. Keep this solution on ice until you are ready to prepare the three enzyme concentrations for the incubations as described in Table S3.
- Vortex again immediately before use. Use within 30 min.

**II. Preparative procedures**

1. **Thermo-shaker temperature checks**

Different instruments may give variations in mixing and temperature. Before starting, it is very important to check that the temperature reached within the Eppendorfs is correct:

- 1. Set the heating block that will be used for the enzyme assay procedure (section III) to 37°C with a stirring rate of 200 rpm.
  2. Prepare 1 Eppendorf (1.5 or 2.0 mL, according to the recommended size for the heating block that will be used) with 1 mL of water.
  3. Monitor the temperature inside the Eppendorf using a thermocouple until equilibrium is reached.
  4. If the temperature of the water inside your Eppendorf tube is not 37°C, adjust the temperature settings accordingly and keep monitoring until an internal temperature of 37°C is reached. Make sure the temperature remains stable over at least 15 minutes (the incubation period of the assay will be 12 minutes).
  5. Record the model of the instrument used as well as the temperature settings on the result sheet.

*Please use the same equipment every time if possible.*

1. **Calibration curve to quantify the liberated maltose**
   1. Pipette 150 µL of each maltose calibrator into safe lock Eppendorf tubes in triplicate. Label with heat and water resistant labels/pen.
   2. Add 75 µL of DNSA reagent solution to each.
   3. Proceed to step 7 in section III.
2. **Moisture content of the starch**
   1. Weigh 3 g of starch (record the exact weight of sample and the weight of the container).
   2. Dry in an oven at 110°C for 18 h.
   3. Equilibrate to room temperature in a desiccator before weighing the sample again.

**III. α-Amylase assay procedure**

1. **Preparation of the α-Amylase assay procedure**
   1. **
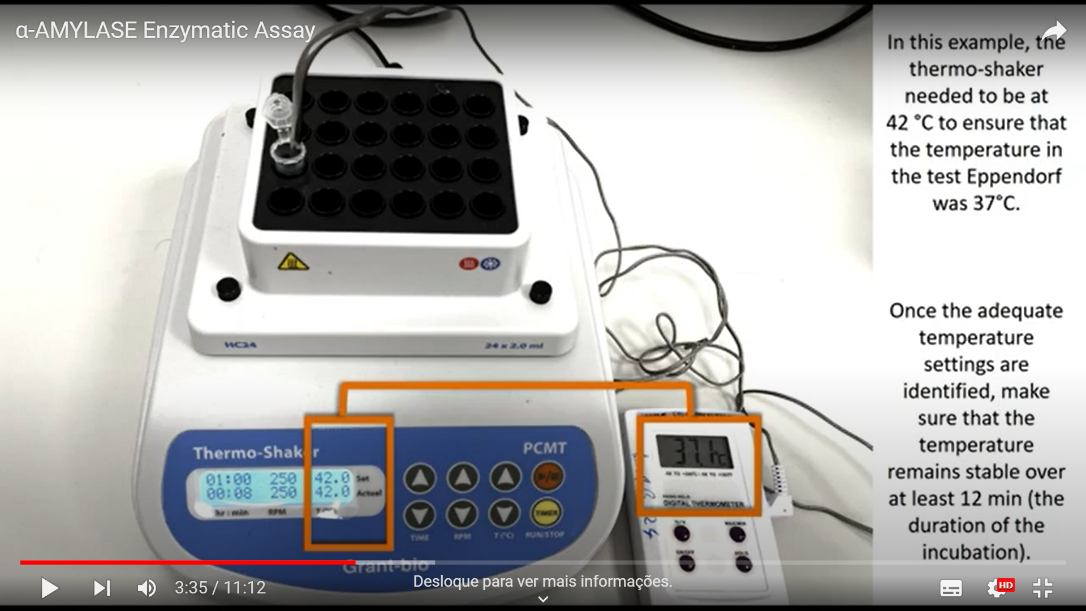
**Set the heating-block to the correct temperature to ensure 37°C inside the Eppendorf tubes.
   2. Place an Eppendorf tube with 1 mL of water in the thermo-shaker to monitor the temperature throughout the experiments using a thermocouple (Picture S2)
   3. Pre-warm the starch solution (but not the enzymes) to 37°C.
   4. Prepare of a polystyrene container with ice.

**Picture S2**- Temperature monitoring during incubations.

- 1. Set a spectrophotometer or microplate reader to 540 nm. Please record the type of instrument used and model in the result sheet.

1. **Prepare the incubation tubes**
   1. Label 3 safe lock Eppendorf tubes (1.5 or 2.0 mL, depending on the appropriate size for the thermo-block that will be used) for the incubations of three concentrations of the α-amylase solution to be tested with the substrate.
   2. Set the Eppendorf tubes in the preheated thermo-shaking block with mixing (250 rpm). Let the temperature equilibrate.
   3. Add 500 µL of pre-warmed potato starch solution (substrate) to each tube.
   4. Maintain the tubes closed until the enzyme is added to prevent evaporation.
2. **Prepare the sample collection tubes**
   1. For each incubation tube, label 4 other safe-lock Eppendorf tubes. These will be used for the samples that will be collected throughout the incubation period.
   2. Add 75 μL of DNSA to each tube.
   3. Keep the pre-filled sample collection tubes closed and protected from light.
3. **Prepare the three enzyme dilutions**
   1. Prepare three enzyme concentrations by diluting your enzyme solution (prepared according to section I.5) in the adequate sodium phosphate buffer as indicated in Table S3.
   2. Keep your enzyme solutions in ice. Mix by inversion immediately before use.

| **Table S3:** Preparation of the enzyme dilutions | | |
| --- | --- | --- |
| **Enzyme solution** | **Volume of mother solution**  **(section I. 5) (mL)** | **Volume of buffer pH 6.9 (mL)** |
| **C1** | 0.650 | 1.350 |
| **C2** | 1 | 1 |
| **C3** | 1.5 | 0.5 |

1. **Start the reactions (⌛ Time-critical steps)**
   1. Add 500 µL of the enzyme concentration to be tested (C1, C2, C3) to the corresponding tube in the thermo-shaker, pre-filled with 500 µL of starch (as shown in **Table S4**):

👍Perform a wet dispense to minimize droplets: Immerse the tip in the solution and dispense directly into the liquid (wet-dispense) to avoid losses through droplet retention on the walls of the Eppendorf.

👍 Mix well through repetitive pipetting: using the same tip, withdraw solution from the tube and dispense it back in (wet-dispense again) two times to ensure adequate mixing.

*🖐 Vortexing is not recommended: higher risk for droplet creation due to the small volumes and possible impact on temperature maintenance*

- 1. Start the timer immediately when the amylase solution is added to the first tube and leave a 30 second interval before each subsequent addition. It is recommended to do a maximum of one set of 3 tubes at one time as the reaction is time sensitive.

1. **Collect samples and stop the enzymatic reaction (⌛ Time-critical steps)**

Take a 150 μL aliquot from each tube at 3, 6, 9 and 12 minutes and transfer immediately to the corresponding sample collection tube pre-filled with DNSA to stop the reaction as shown in **Table S5**. **Take each aliquot as close as possible to its respective sample collection time, within a maximum of ± 5 seconds.**

Follow the practices described in 5.1:

👍Perform a wet dispense into the DNSA pre-filled tubes to minimize droplets

👍 Mix well through repetitive pipetting

1. **Prepare the samples for the absorbance measurements**
   1. Set the thermo-shaker to 100°C.
   2. Place your samples and calibrators in a microfuge for 30 seconds or centrifuge (1000 *g*, 2 min) so that all droplets are brought back into solution.
   3. Place the samples and calibrators in the thermo-shaker (100°C, 15 minutes) and then transfer them to the icebox to cool for 15 minutes.
   4. Add 675 µL of purified water to each tube and mix by inversion.
   5. Transfer the samples and calibrators to a cuvette or pipette to a microtiter plate (300 µL per well) and record the absorbance at 540 nm.

| **Table S4: Enzymatic reaction tubes.** | | | | |
| --- | --- | --- | --- | --- |
|  | **Enzyme concentration C1** | **Enzyme concentration C2** | **Enzyme concentration C3** |
| **Substrate: Potato starch solution (μL)** | 500 | 500 | 500 |
| **α-amylase (μL)** | 500 | 500 | 500 |
| **Total volume at t_0_ (μL)** | 1000 | 1000 | 1000 |

| **Table S5: Sample collection tubes** | | | |
| --- | --- | --- | --- |
|  | **Sample collection tubes for Enzyme concentration C1** | **Sample collection tubes for Enzyme concentration C2** | **Sample collection tubes for Enzyme concentration C3** |
| **Colour reagent (μL)** | 75 | 75 | 75 |
| **Sample (μL)** | 150 | 150 | 150 |
| ***30 s spin in microfuge or light centrifugation (1000 g, 2 min)*** | | | |
| ***Incubate at 100°C for 15 minutes*** | | | |
| **Purified water (μL)** | 675 | 675 | 675 |
| **Final volume (μL)** | 900 | 900 | 900 |

The enzyme concentrations recommended in section I.5 have been tested and resulted in maltose concentrations within the range of the calibration curve. The results obtained for all enzymes over the 12 minute incubations were also within a linear range with R-squared values between 0.95 and 0.99.

For reference purposes, the range of maltose concentrations obtained at each incubation temperature is presented below:

Incubations at 37°C – Lowest initial maltose concentrations were around 0.5 mg/mL (3 min of incubation with enzyme concentration C1). With the highest enzyme concentrations (concentration C3) the final maltose concentrations were around 2-2.5 mg/mL (after 12 min of incubation with concentration C3) depending on the enzyme.

Incubations at 20°C - Initial maltose concentrations were around 0.5 mg/mL (3 min of incubation with enzyme concentration C1). With the highest enzyme concentrations (concentration C3) final maltose concentrations ranged between 1.2 and 2 mg/mL (after 12 min of incubation with concentration C3) depending on the enzyme.

Some degree of variability is expected between labs due to, for example some level of reduction in enzyme activity due to temperature differences during shipping. However, some level of enzymatic activity would still be expected at the recommended enzyme concentrations. If no enzymatic activity is detected at these concentrations, it may be necessary to check all the critical steps in the protocol and repeat the experiments.

**IV. Calculations**

Please use the excel file provided in the google drive to report your data. This section describes the calculations used in this excel file. When completing your excel file:

- Fill in the yellow cells
- Do not change the formulas in the other cells
- If changes are needed, please highlight any changes made to the spreadsheets by using the comment function

1. **Calibration Curve**
   1. Subtract the colour reagent blank from all readings.
   2. Plot the concentration of the maltose calibrators (mg) against the corresponding ΔA_540nm_.
   3. Establish the linear regression (only use the linear part of the curve > the calibration blank should not be included as a data point in the calibration curve):

m: slope

b: intercept with y axis

1. **Calculation of enzyme activity (based on the slope of the linear trendline)**
   1. Subtract the absorbance of the colour reagent blank (section IV.0) from all readings.
   2. Use the maltose calibration curve to calculate the maltose concentration (mg/mL) reached with each enzyme concentration at each incubation time

: slope of the calibration curve

: intercept with y axis of the calibration curve

- 1. For each enzyme concentration, plot the maltose concentration (mg/mL) against time (*t*_min_) and establish the corresponding linear regression (only use the linear part of the curve):

: slope (maltose concentration/min)

$b$: intercept with y axis

- 1. For each enzyme concentration, calculate the enzyme concentration (mg/mL for enzyme powders, or µL/mL if using saliva (mL) in the incubation mixture. Example for enzyme concentration C1:

$$Enzyme concentration during incubations=\frac{\left( 0.650\times Cenzyme \right)}{2} \times0.5\div1$$

0.650: volume (mL) of enzyme solution used in preparing concentration A

$Cenzyme$: concentration of enzyme solution prepared according to section I.5

$2$: volume of solution prepared to obtain concentration A

$0$.5: volume of enzyme solution (mL) used during the incubation

1: incubation volume (mL)

- 1. For each enzyme concentration, units of enzyme can be calculated using the following equation

$$Activity (U per mg or \mu L of enzyme product)= 3min\times\frac{Slope as calculated in 9.3 (\frac{maltose concentration (\frac{mg}{mL})}{min})}{Enxyme concentration during incubations as calculated in 9.4 \left( \frac{mg}{mL} or\frac{\mu L}{mL} \right)}$$

***Unit definition:*** *One unit liberates 1.0 mg of maltose from starch in 3 minutes at pH 6.9 at 37°C.*

# Results obtained during preliminary tests

## Comparison of the two DNSA lots shipped to the participating laboratories

Prior to shipping, calibration curves established with solutions prepared from each of these lots have been compared and showed very similar results. These results are presented in Figure S1.

**Figure S1** – Calibration curves established with DNSA colour reagents prepared from two different DNSA lots (lots MKCH6290 and MKBR3017V). Each point represents the average of three repetitions (on three different days) ± SD (except for the fourth point referring to lot MKCH6290 for which the mean of two repetitions only is presented)

## Examples of nonlinear reaction curves

Prior to shipping, all products were tested at different concentrations to identify the concentrations within the linear activity range. Examples of nonlinear reaction curves obtained with saliva solutions are presented in Figure S2.

**Figure S2** – Examples of nonlinear reaction curves obtained with saliva solutions that were too concentrated.

# Results obtained during the ring trial

## Implementation of the protocol by the participating laboratories

| **Table S6** – Instruments used by each lab for the incubations and absorbance measurements | | | | | |
| --- | --- | --- | --- | --- | --- |
|  | **Incubations** | | | **Absorbance measurements** | |
|  | **Instrument model (Brand, Country)** | **Type of instrument** | **Shaking during incubations** | **Instrument model**  **(Brand, Country)** | **Type of instrument** |
| **Lab A** | Grant-Bio PCMT Thermoshaker  (Grant Instruments, United Kingdom) | Thermal mixer | Yes | SpectraMax ABS Plus (Molecular Devices, United States) | Microplate |
| **Lab B** | Julabo Corio CD  (Julabo GmbH, Germany) | Water bath | No | Thermo Scientific™ Multiskan™ GO Microplate Spectrophotometer  (Thermo Fisher Scientific, United States) | Microplate |
| **Lab C** | Eppendorf ThermoMixer® C (Eppendorf, Germany) | Thermal mixer | Yes | SPECTROstar Nano  (BMG Labtech, Germany) | Microplate |
| **Lab D** | Eppendorf ThermoMixer® C (Eppendorf, Germany) | Thermal mixer | Yes | VersaMax Microplate Reader (Molecular Devices, United States) | Microplate |
| **Lab E** | Eppendorf ThermoMixer® C  (Eppendorf, Germany) | Thermal mixer | Yes | BMG Labtech CLARIOstar Version 5.70 R3  (BMG Labtech, Germany) | Microplate |
| **Lab F** | TRM 750  (Asal S.r.l., Italy) | Water bath | Yes | Shimadzu UV-1800 Spectrophotometer  (Shimadzu Corporation, Japan) | Cuvette |
| **Lab G** | DC30  (Thermo Haake, United States) | Water bath | No | Shimadzu UV-1800 Spectrophotometer  (Shimadzu Corporation, Japan) | Cuvette |
| **Lab H** | Eppendorf ThermoMixer® C  (Eppendorf, Germany) | Thermal mixer | Yes | Spark® Multimode Microplate Reader  (Tecan, Switzerland) | Microplate |
| **Lab I** | IKA C-MAG HS 7  (IKA-Werke GmbH & Co. KG, Germany) | Water bath | No | Shimadzu UV 2501PC Spectrophotometer (Shimadzu Corporation, Japan) | Cuvette |
| **Lab J** | Eppendorf ThermoMixer® C  (Eppendorf, Germany) | Thermal mixer | Yes | Spark® Multimode Microplate Reader  (Tecan, Switzerland) | Microplate |
| **Lab K** | Eppendorf ThermoMixer® Comfort  (Eppendorf, Germany) | Thermal mixer | Yes | Biotek Synergy 2 Plate Reader (BioTek Instruments – Agilent Technologies, United States) | Microplate |
| **Lab L** | Eppendorf ThermoMixer® C  (Eppendorf, Germany) | Thermal mixer | No | Microplate Reader  (Thermoscientific, USA) | Microplate |
| **Lab M** | Eppendorf ThermoMixer® C  (Eppendorf, Germany) | Thermal mixer | Yes | Beckman Coulter DU800  (Beckman Coulter, Inc., United States) | Cuvette |

|  | **Table S7** – Recommended and tested concentrations of the enzyme solutions used in the preparation of diluted test solutions C1, C2 and C3 for incubations at 37°C. | | | | | | | |
| --- | --- | --- | --- | --- | --- | --- | --- | --- |
|  | | **Recommended concentrations** | **Tested concentrations** | | | | | |
|  | | (µg/mL for powder preparations or µL/mL for saliva) | Mean ± SD (n)  (µg/mL for powder preparations or µL/mL for saliva) | | | Mean ± SD (n)  (U/mL)^1^ | | |
| **α-Amylase M** | | 2.7 | 2.7 | ± | <0.1 (13) | 1.1 | ± | 0.2 (12) |
| **α-Amylase S** | | 40 | 41.8 | ± | 5.7 (13) | 0.9 | ± | 0.3 (12) |
| **Pancreatin** | | 4 | 4.1 | ± | 0.1 (13) | 0.8 | ± | 0.1 (12) |
| **Human Saliva** | | 1.2 | 1.2 | ± | <0.1 (13) | 1.1 | ± | 0.2 (13) |
|  | ^1^Calculation of mean concentrations in U/mL based on respective enzyme activities at 37°C, outlier data points excluded from the calculations | | | | | | | |

| **Table S8 –** Calibration curves. | | | | | | | | | | | | | | | | | | | | | | | | | | | | | | | | |
| --- | --- | --- | --- | --- | --- | --- | --- | --- | --- | --- | --- | --- | --- | --- | --- | --- | --- | --- | --- | --- | --- | --- | --- | --- | --- | --- | --- | --- | --- | --- | --- | --- |
| **Panel A -** Values of absorbance measured at 540 nm as reported by each participating laboratory. | | | | | | | | | | | | | | | | | | | | | | | | | | | | | | | | |
| **Laboratory** | **A** | | | **B** | **C** | **D** | | | | **E** | | **F** | | **G** | | | **H** | | | | **I** | **J** | | **K** | **L** | | | | **M** | | | |
| **Calibration curve number** | **1** | **2** | **3** | **4** | **5** | **6** | **7** | **8** | **9** | **10** | **11** | **12** | **13** | **14** | **15** | **16** | **17** | **18** | **19** | **20** | **21** | **22** | **23** | **24** | **25** | **26** | **27** | **28** | **29** | **30** | **31** | **32** |
| **Maltose concentration**  **(mg/mL)** | **Absorbance at 540 nm** | | | | | | | | | | | | | | | | | | | | | | | | | | | | | | | |
| 0.0 | 0.058 | 0.052 | 0.055 | 0.058 | 0.050 | 0.052 | 0.051 | 0.050 | 0.052 | 0.053 | 0.056 | 0.171 | 0.180 | 0.071 | 0.071 | 0.073 | 0.199 | 0.204 | 0.207 | 0.205 | 0.145 | 0.058 | 0.054 | 0.087 | 0.062 | 0.059 | 0.061 | 0.063 | 0.044 | 0.044 | 0.054 | 0.054 |
| 0.2 | 0.107 | 0.105 | 0.104 | 0.096 | 0.113 | 0.121 | 0.115 | 0.112 | 0.122 | 0.061 | 0.059 | 0.176 | 0.256 | 0.122 | 0.120 | 0.119 | 0.228 | 0.223 | 0.221 | 0.221 | 0.248 | N/A | 0.114 | 0.132 | 0.122 | 0.118 | 0.134 | 0.117 | 0.059 | 0.060 | 0.066 | 0.067 |
| 0.4 | 0.230 | 0.206 | 0.201 | 0.181 | 0.183 | 0.208 | 0.203 | 0.203 | 0.211 | 0.138 | 0.152 | 0.278 | 0.382 | 0.225 | 0.222 | 0.220 | 0.306 | 0.297 | 0.295 | 0.291 | 0.323 | N/A | 0.200 | 0.198 | 0.239 | 0.239 | 0.240 | 0.228 | 0.118 | 0.112 | 0.124 | 0.120 |
| 0.6 | 0.319 | 0.301 | 0.311 | 0.285 | 0.281 | 0.347 | 0.298 | 0.307 | 0.350 | 0.232 | 0.266 | 0.372 | 0.497 | 0.323 | 0.329 | 0.325 | 0.379 | 0.379 | 0.384 | 0.374 | 0.432 | 0.318 | 0.296 | 0.301 | 0.365 | 0.365 | 0.365 | 0.346 | 0.184 | 0.202 | 0.179 | 0.184 |
| 0.8 | 0.442 | 0.422 | 0.415 | 0.405 | 0.386 | 0.421 | 0.405 | 0.425 | 0.426 | 0.287 | 0.297 | 0.463 | 0.628 | 0.438 | 0.432 | 0.431 | 0.443 | 0.439 | 0.434 | 0.441 | 0.565 | 0.428 | 0.383 | 0.374 | 0.487 | 0.493 | 0.483 | 0.461 | 0.257 | 0.257 | 0.251 | 0.248 |
| 1.0 | 0.558 | 0.534 | 0.518 | 0.499 | 0.490 | 0.631 | 0.543 | 0.572 | 0.637 | 0.365 | 0.395 | 0.642 | 0.750 | 0.536 | 0.539 | 0.529 | 0.523 | 0.523 | 0.528 | 0.518 | 0.682 | 0.536 | 0.489 | 0.504 | 0.606 | 0.641 | 0.609 | 0.570 | 0.320 | 0.323 | 0.316 | 0.320 |
| 1.5 | 0.809 | 0.802 | 0.782 | 0.753 | 0.745 | 0.834 | 0.771 | 0.796 | 0.842 | 0.520 | 0.583 | 0.935 | 1.068 | 0.843 | 0.858 | 0.835 | 0.670 | 0.679 | 0.689 | 0.677 | 0.900 | 0.789 | 0.713 | 0.706 | 0.916 | 0.959 | 0.917 | 0.874 | 0.508 | 0.502 | 0.482 | 0.485 |
| 2.0 | 1.083 | 1.083 | 1.037 | 1.007 | 0.996 | 1.309 | 1.227 | N/A | 1.322 | 0.896 | 0.776 | 1.256 | 1.367 | 1.111 | 1.113 | 1.090 | 0.856 | 0.866 | 0.885 | 0.857 | 1.325 | 1.033 | 0.956 | 0.883 | 1.234 | 1.257 | 1.227 | 1.163 | 0.685 | 0.690 | 0.648 | 0.652 |
| 2.5 | N/A | N/A | N/A | 1.218 | 1.251 | 1.502 | 1.446 | 1.477 | 1.513 | 1.171 | 1.037 | 1.511 | 1.642 | 1.366 | 1.372 | 1.349 | 1.020 | 1.033 | 1.055 | 1.024 | 1.600 | 1.315 | 1.185 | 1.164 | 1.525 | 1.593 | 1.534 | 1.468 | 0.879 | 0.879 | 0.829 | 0.828 |
| 3.0 | N/A | N/A | N/A | 1.442 | 1.490 | 2.111 | 1.824 | 1.772 | 2.125 | 1.370 | 1.336 | 1.835 | 1.913 | 1.590 | 1.632 | 1.595 | 1.224 | 1.223 | 1.243 | 1.203 | 1.848 | 1.580 | 1.436 | 1.366 | 1.841 | 1.928 | 1.816 | 1.770 | 1.052 | 1.052 | 0.989 | 0.990 |
| **Panel B –** Slopes and r^2^ of calibration curves^1^ (determined after subtracting the blank absorbance from each individual absorbance value) | | | | | | | | | | | | | | | | | | | | | | | | | | | | | | | | |
| **Slope** | 1.85 | 1.83 | 1.92 | 2.05 | 2.00 | 1.44 | 1.62 | 1.66 | 1.43 | 2.06 | 2.28 | 1.66 | 1.67 | 1.86 | 1.83 | 1.87 | 2.87 | 2.83 | 2.76 | 2.87 | 1.68 | 1.91 | 2.12 | 2.25 | 1.63 | 1.55 | 1.64 | 1.69 | 2.78 | 2.79 | 3.00 | 2.99 |
| **r^2^** | 1.00 | 1.00 | 1.00 | 1.00 | 1.00 | 0.98 | 0.99 | 1.00 | 0.98 | 0.99 | 0.99 | 1.00 | 1.00 | 1.00 | 1.00 | 1.00 | 1.00 | 1.00 | 1.00 | 1.00 | 1.00 | 1.00 | 1.00 | 1.00 | 1.00 | 1.00 | 1.00 | 1.00 | 1.00 | 1.00 | 1.00 | 1.00 |
| **Panel C –** Average of the slopes and r^2^ of calibration curves (split according to the type of instrument used for the absorbance measurements) | | | | | | | | | | | | | | | | | | | | | | | | | | | | | | | | |
|  | **Microplate**  (n=22 calibration curves) | | | | | | **Cuvette**  (n = 10 calibration curves) | | | | | |  | | | | | | | | | | | | | | | | | | | |
|  | **Average** ± **SD** | | | **CV_R_ (%)** | **Min** | **Max** | **Average** ± **SD** | | | **CV_R_ (%)** | **Min** | **Max** |
| **Slope** | 2.01 ± 0.46 | | | 23 | 1.43 | 2.87 | 2.21 ± 0.59 | | | 27 | 1.66 | 3.07 |
| **r^2^** | 1.00 ± 0.01 | | | 1 | 0.98 | 1.00 | 1.00 ± 0.00 | | | 0 | 1.00 | 1.00 |
| ^1^Highligted cells denote results from calibration curves established using the cuvette format | | | | | | | | | | | | | | | | | | | | | | | | | | | | | | | | |

## Calibration curves

## Method’s performance – complementary measurements

The bias scores used in the calculation of the Z-scores for the results obtained at 37°C are presented in Figure S3. The bias scores conversion to Z-scores for the results for the results obtained at 20°C are presented in Figure S4 (panels a and b, respectively).

**Figure S3** – Bias scores (%) of each laboratory's using the mean of all laboratories as reference, for each product assayed at 37°C.

**(a)**

**(b)**

Figure S4. Bias Z-score analysis for the results obtained at 20°C. (a) Bias scores (%) of each laboratory using the mean of all laboratories as reference, for each product. (b) Z-score distribution of each laboratory's bias relative to the mean of all laboratories, for each product. Each point represents one product by an individual laboratory. Horizontal line indicates reference (z = 0), highlighted area between |z| ≤ 2 indicates satisfactory performance, areas located at |z| ≤ 3 indicate unsatisfactory performance.

1. Teagasc Food Research Centre, Moorepark, Fermoy, Co Cork P61 C996, Ireland [↑](#footnote-ref-2)
2. INRAE, Institut Agro, STLO, 35042 Rennes, France [↑](#footnote-ref-3)
3. Laboratory of Food Chemistry and Biochemistry, Department of Food Science and Technology, School of Agriculture, Aristotle University of Thessaloniki, P.O. Box 235, 54124, Thessaloniki, Greece [↑](#footnote-ref-4)
4. School of Food Science and Nutrition, University of Leeds, Leeds, United Kingdom [↑](#footnote-ref-5)
5. Laboratory of Food Technology, Department of Microbial and Molecular Systems (M2S), KU Leuven, Kasteelpark Arenberg 23, PB 2457, 3001, Leuven, Belgium [↑](#footnote-ref-6)
6. School of Biosciences, Faculty of Health and Medical Sciences, University of Surrey, Guildford, GU2 7XH, United Kingdom [↑](#footnote-ref-7)
7. Center for Innovative Food (CiFOOD), Department of Food Science, Aarhus University, Agro Food Park 48, Aarhus N 8200, Denmark [↑](#footnote-ref-8)
8. Department of Horticulture, Martin-Gatton College of Agriculture, Food and Environment, University of Kentucky, Lexington, Kentucky, USA [↑](#footnote-ref-9)
9. Department of Agricultural, Food, Environmental and Animal Sciences, University of Udine, Italy [↑](#footnote-ref-10)
10. Food Technology Area, Department of Agricultural Engineering, University of Valladolid, Spain [↑](#footnote-ref-11)
11. Department of Clinical Nutrition, Faculty of Applied Medical Sciences, King Abdulaziz University, Jeddah, Saudi Arabia [↑](#footnote-ref-12)
12. Wageningen Food & Biobased Research, Wageningen University & Research, 6708 WG Wageningen, The Netherlands [↑](#footnote-ref-13)
13. Quadram Institute Bioscience, Rosalind Franklin Road, Norwich Research Park, Norwich, NR4 7UQ, United Kingdom [↑](#footnote-ref-14)
14. Department of Food Engineering, Faculty of Engineering, Ege University, 35100, İzmir, Türkiye [↑](#footnote-ref-15)
15. School of Food Science and Engineering, South China University of Technology, Guangzhou 510640, China [↑](#footnote-ref-16)
16. Global Oatly Science and Innovation Centre, Rydbergs Torg 11, Space Building, Science Village, 22 484 Lund, Sweden [↑](#footnote-ref-17)
17. Nofima AS, Norwegian Institute of Food, Fisheries and Aquaculture Research, PB 210, N-1433, Ås, Norway [↑](#footnote-ref-18)
18. The calculations for the buffer preparation are dependent on the hydration status of the salts. If you use a different hydration status, adjust the weight of the salt according to the molecular weight. [↑](#footnote-ref-19)
19. Measure the moisture content of the starch and maltose prior to use, and record the moisture content. If available, it is strongly recommended to store starch and maltose under desiccator conditions to avoid moisture absorption. If moisture >5%, desiccate under vacuum prior to use. [↑](#footnote-ref-20)
